# Locally Fixed Alleles: A method to localize gene drive to island populations

**DOI:** 10.1038/s41598-019-51994-0

**Published:** 2019-11-01

**Authors:** Jaye Sudweeks, Brandon Hollingsworth, Dimitri V. Blondel, Karl J. Campbell, Sumit Dhole, John D. Eisemann, Owain Edwards, John Godwin, Gregg R. Howald, Kevin P. Oh, Antoinette J. Piaggio, Thomas A. A. Prowse, Joshua V. Ross, J. Royden Saah, Aaron B. Shiels, Paul Q. Thomas, David W. Threadgill, Michael R. Vella, Fred Gould, Alun L. Lloyd

**Affiliations:** 1 Department of Mathematics, North Carolina State University, Raleigh, NC 27695 USA; 2 Biomathematics Graduate Program, North Carolina State University, Raleigh, NC 27695 USA; 3 Department of Biological Sciences, North Carolina State University, Raleigh, NC 27695 USA; 4 Island Conservation, 2100 Delaware Ave., Suite 1, Santa Cruz, CA 95060 USA; 5 Department of Entomology and Plant Pathology, North Carolina State University, Raleigh, NC 27695 USA; 6 National Wildlife Research Center, US Department of Agriculture, Fort Collins, CO 80521 USA; 7 CSIRO Land & Water, Centre for Environment and Life Sciences, Floreat, WA Australia; 8 Genetic Engineering and Society Center, North Carolina State University, Raleigh, NC 27695 USA; 9 Department of Microbiology, Immunology & Pathology, Colorado State University, Fort Collins, CO 80523 USA; 10 School of Mathematical Sciences, The University of Adelaide, Adelaide, SA 5005 Australia; 11 The Robinson Research Institute and School of Medicine, The University of Adelaide, Adelaide, SA 5005 Australia; 12 Department of Molecular and Cellular Medicine, Texas A&M University, College Station, TX 77843 USA

**Keywords:** Evolutionary ecology, Evolutionary genetics, Ecological modelling

## Abstract

Invasive species pose a major threat to biodiversity on islands. While successes have been achieved using traditional removal methods, such as toxicants aimed at rodents, these approaches have limitations and various off-target effects on island ecosystems. Gene drive technologies designed to eliminate a population provide an alternative approach, but the potential for drive-bearing individuals to escape from the target release area and impact populations elsewhere is a major concern. Here we propose the “Locally Fixed Alleles” approach as a novel means for localizing elimination by a drive to an island population that exhibits significant genetic isolation from neighboring populations. Our approach is based on the assumption that in small island populations of rodents, genetic drift will lead to alleles at multiple genomic loci becoming fixed. In contrast, multiple alleles are likely to be maintained in larger populations on mainlands. Utilizing the high degree of genetic specificity achievable using homing drives, for example based on the CRISPR/Cas9 system, our approach aims at employing one or more locally fixed alleles as the target for a gene drive on a particular island. Using mathematical modeling, we explore the feasibility of this approach and the degree of localization that can be achieved. We show that across a wide range of parameter values, escape of the drive to a neighboring population in which the target allele is not fixed will at most lead to modest transient suppression of the non-target population. While the main focus of this paper is on elimination of a rodent pest from an island, we also discuss the utility of the locally fixed allele approach for the goals of population suppression or population replacement. Our analysis also provides a threshold condition for the ability of a gene drive to invade a partially resistant population.

## Introduction

Genetic modification of pest species has been suggested as a means to address a wide variety of pest problems, including those impacting human health, pre- and post-harvest crop losses, and conservation of endangered species^1–5^. One approach to genetic pest management involves introducing a DNA sequence into the pest genome that causes its own over-representation in future generations by inducing super-Mendelian inheritance; generally referred to as gene drive. An engineered gene coding for a desirable trait, e.g. one that renders the pest species less troublesome (e.g. reduction of vector competence of a mosquito species) can be linked to the gene drive sequence in order to increase its frequency in the population. Alternatively, the gene drive sequence can be engineered to disrupt a gene critical to fitness, or to deliver a payload that achieves this aim, and thereby suppress, or even eliminate, a population.

Ever since such drives were proposed, concerns have been raised in the peer-reviewed literature and in the popular media about unintended consequences of releases and ethical dimensions of the work. These include failure of the genetic construct being driven, but also the spread of gene drives beyond the region in which spread is intended and approved by the local population and governing authorities^2–13^. The nature and seriousness of these concerns differ between different gene drive technologies and applications (for instance, considerations would be quite different for an approach intended to replace a human disease-vectoring mosquito species by a variant that is refractory to the pathogen than for removal of an invasive species that causes ecological damage). These concerns are most acute in the case of drives that are designed to suppress and eliminate a population: spread of such a drive could have a risk of leading to global eradication of a species that is a pest in one area but a valuable component of an ecosystem in other areas (e.g. mice and rats). As a result, there is much interest in the ability to design gene drives that exhibit spatial localization, i.e. ones that have the ability to spread in a given region but will not spread globally.

Several approaches have been suggested to achieve localization of gene drives. Drives that exhibit an invasion threshold, such as the engineered underdominance (EU) approach, provide a natural means to achieve localization^14,15^. These drives exhibit frequency-dependent dynamics where the drive can only spread if its frequency exceeds a particular level—the invasion threshold. Below this level, the frequency of the drive will decrease, leading to its loss from the population. Spread of a threshold drive across a patchy environment is more difficult, and becomes highly unlikely or even impossible when the invasion threshold is 50% or higher^16,17^. Other approaches have been suggested to achieve localization, including killer-rescue^18^, multi-locus assortment^19^, sex-linked genome editors^20^ and daisy-chain drive^21^. Theoretical analysis has suggested the ability of daisy-chain drive to simultaneously achieve spread and localization to a single area is only possible in a limited set of circumstances, and this concern also pertains to some other gene drives developed to be localized^22^. While all of these localized drives could change characteristics of pests in a population, their ability to locally suppress populations is questionable^22,23^, although see also^24^.

In this paper, we propose a localization method, the “Locally Fixed Alleles” (LFA) approach, that can be utilized for relatively small populations that exhibit a significant degree of genetic isolation from other populations. This method is particularly suited for elimination of pest species from small oceanic islands, where the target population has small effective population size and for which there is naturally limited gene flow with other populations. While multiple alleles are expected to be commonly maintained at loci in large populations, genetic drift in small island populations is predicted to result in fixation of alleles at some loci in the genome^25,26^.

Utilizing the high degree of genetic specificity of homing drives based on the CRISPR/Cas9 system^4^, our approach aims at employing one or more locally fixed alleles as the target for a gene drive on a particular island. Such a drive can spread to individuals carrying that allele, but individuals that do not have that specific allele are naturally resistant to the drive. For example, polymorphisms that occur in targeted Cas9 guide RNA binding sites or protospacer adjacent motifs (PAM) may effectively limit gene drive activity^27^ such that a drive can spread to individuals carrying alleles that form functional sites, but individuals with alternate alleles are naturally resistant to the drive. By design, we would search for alleles that are fixed in the population on the target island but not fixed in populations beyond that island. Consequently, the drive could be expected to result in only limited transient suppression beyond the island. A special case of this approach, the “private allele” (PA) approach (dubbed “precision drive” by Esvelt *et al*.^4^; see also^12^), occurs when the target allele is specific to the target population, but absent from other populations. We emphasize that the LFA approach does not require the target allele be a private allele, simply that it not be fixed in non-target populations.

The LFA approach can be used with a variety of different gene drives (e.g. standard homing drives, sex-biasing drives, and so on). Here, for simplicity, we illustrate the method using a standard homing drive^2,4^ aimed at population elimination. We describe this in the setting of removal of a rodent species, such as the house mouse, *Mus musculus*, from an island. Invasive mice, and other rodents, are a particular concern for species conservation^28^, having significantly impacted many island ecosystems, including causing extinctions of endemic island vertebrate, invertebrate and plant species^29–31^. Although an island release would involve procedures that attempt to confine the gene drive mice to the island, unintended escape of these mice must be considered a possibility. Here, we use mathematical modeling to explore the impact that escape of drive individuals would have on mainland populations.

While the focus of this study is on a drive that can eliminate a rodent pest species from an island, the LFA approach can be used more generally for drives aimed at population suppression or replacement provided that the drive bears some fitness cost. These more general settings are discussed in detail in the Supplementary Information, including some important differences in the dynamics from the elimination setting discussed in the main text.

This paper is organized as follows: we first introduce the mathematical model, then briefly describe the single-patch (island-only) dynamics before discussing those seen in a two-patch (island-mainland) setting. Supplementary information includes the derivation of an analytic threshold condition for the ability of drive to invade a partially susceptible population, an exploration of the sensitivity of results to various drive and ecological/demographic parameters, initial results of a stochastic model for the dynamics of LFA, and discussion of the use of LFA in more general population suppression and replacement settings.

## Methods

We employ a continuous-time non age-structured island-mainland model that describes the population dynamics and genetics of two populations. As our primary concern here is the impact of escape from the island, we assume unidirectional migration from the island to the mainland. (We recognize that migration from mainland to island would be an important consideration in the period following successful suppression or eradication from the island, but this is not the topic of this study.) Throughout, we assume a 50:50 sex ratio and so track numbers of female individuals. For an *n*-genotype system, denoting genotypes with a subscript and denoting island population numbers with superscript *I* (*N*^*I*^_*i*_) and mainland population numbers with a superscript *M* (*N*^*M*^_*i*_), we have

1 $$\frac{d{N}_{i}^{I}}{dt}={f}^{I}({N}_{1}^{I},\cdots ,{N}_{n}^{I})-\mu {N}_{i}^{I}$$

2 $$\frac{d{N}_{i}^{M}}{dt}={f}^{M}({N}_{1}^{M},\cdots ,{N}_{n}^{M})+\mu {N}_{i}^{I}.$$

Here, the functions *f* ^*I*^ and *f* ^*M*^ describe the population dynamics and genetics that occur on island and mainland, respectively, and we assume unidirectional migration with per-capita migration rate equal to µ.

We assume that island and mainland populations both undergo random mating (i.e. are well-mixed) and exhibit logistic-type population dynamics. Our description of population dynamics is based on an earlier model^32^ for the population genetics and dynamics of gene drive in an island mouse population. Per-capita birth and death rates both change linearly with population size, with different coefficients on mainland and island (see^32^ and references therein). Within either the island or mainland, and in the absence of migration, genotype dynamics are described by

3 $$\frac{d{N}_{i}}{dt}={b}_{i}(t)max{\textstyle (}1-q\sum _{j}{N}_{j}\,,\,0{\textstyle )}-\rho {N}_{i}-\alpha {N}_{i}\sum _{j}{N}_{j}.$$

Here, superscripts denoting the location have been suppressed for clarity on both state variables and parameters. The functions *b*_*i*_(*t*), described below, depict the genotype-specific birth rates in the absence of density dependence. For an entirely wild-type population, *b*(*t*) would equal *λN*, where *λ* is the per-capita fecundity rate. The *b*_*i*_(*t*) are multiplied by a function that describes the linear density-dependent decline in per-capita birth rates with total population size. (Note that the max function is required to ensure that birth rates remain non-negative.) Per-capita death rates are assumed to increase linearly with overall population size but be independent of genotype. The density-independent component of the per-capita death rate (i.e. the reciprocal of the average lifespan when the population is at low density) is written as ρ, while the coefficient α describes the density-dependent linear increase in per-capita mortality. With these population dynamics, a single patch has a wild-type carrying capacity of $N=\rho ({R}_{0}-1)/(\lambda q+\alpha )$, where the basic reproductive number, *R*_0_, (i.e. the average number of female offspring of a female over its lifetime, at low population density) is equal to *λ*/ρ.

Population genetics is determined by the functions *b*_*i*_(*t*) which give the genotype-specific birth rates (c.f. the model of Robert *et al*.^33^) before accounting for the effects of density-dependence

4 $${b}_{i}(t)=\lambda {w}_{i}\sum _{j}\sum _{k}\frac{P(i|j,k){N}_{j}{N}_{k}}{{\sum }_{l}{N}_{l}}.$$

Here, *λ* is the baseline per-capita birth rate for females. P(*i*| *j*,*k*) gives the proportion of offspring from a mating involving individuals of types *j* and *k* that will have genotype *i*. The effects of the gene drive on biasing of inheritance are coded into these quantities. (The 216 entries of *P*(*i*| *j*,*k*), together with a fully written out set of model equations, appear in the Maple worksheet in the Supplementary Information.) The *w*_*i*_ describe genotype-specific relative fitnesses, which we assume here to act at the embryonic stage and be equal in males and females of a given genotype.

As mentioned above, we use a simple homing-based elimination drive to illustrate the LFA method. We consider three alleles: the drive allele (D), the susceptible (S) allele, i.e. the target for the drive, and an allele that is resistant to the drive (R). All individuals in the target (island) population initially have the genotype SS, but those in the non-target (mainland) population can have SS, RS or RR genotypes. We assume only a single resistance allele in the mainland population. A large mainland population could have a number of different alleles that would be resistant to the drive, but they would all act similarly in being unaffected by the drive. Therefore our 3-allele model is sufficient for capturing dynamics of these cases. Note that here we are considering natural resistance to the drive, rather than drive-resistant alleles that are generated de novo as a result of the drive, e.g. by non-homologous end joining during homing. As a consequence, our model over-estimates the ability of the drive to spread and suppress populations^34^. Given that we are trying to evaluate the risk and implications of escape of drive, this assumption is conservative for our purposes. Homing is assumed to occur during gametogenesis, meaning that successful homing leads to an SD heterozygote individual giving rise to only D gametes. (Homing exclusively in the germline at any point during development would have the same consequences.) Successful homing occurs with probability *e*. The fitness, *w*_*i*_, of SS, RR, or SR individuals is assumed to be 1. The fitness of DD individuals is (1-*s*), and the fitness of SD and RD individuals is (1-*hs*), where *s* is the fitness cost of the drive and *h* is the degree of dominance of the fitness cost. For instance, a recessive lethal drive has *s* = 1 and *h* = 0. Notice that we assume there is no fitness cost for the RR individuals that occur naturally in the non-target population.

We employ parameters (see Table 1) that are appropriate for *Mus musculus* populations, largely based on those used by Backus & Gross^32^ (see also^35^). For this set of parameters, the basic reproductive number of a wild-type population is 3.5. Assuming an island of area 6 hectares, these parameters lead to an equilibrium population size of 1000 females. We assume that the mainland has a population size that is 100 times larger than this.

**Table 1:** List of Parameters and Their Values.

	Parameter	Value	Source
*λ*	Female fecundity rate	8.4 (year)^−1^	Backus & Gross^32^
*q*	Coefficient quantifying density-dependent decline in birth rate	Island: 6.427 × 10^−3^ (mouse)^−1^ Mainland: 6.427 × 10^−3^ (mouse)^−1^	Backus & Gross^32^
*ρ*	Density independent per-capita death rate	2.4 (year)^−1^	Backus & Gross^32^
*α*	Coefficient quantifying density-dependent increase in death rate	Island: 5.000 × 10^−5^ (mouse)^−1^ (year)^−1^ Mainland: 5.000 × 10^−7^ (mouse)^−1^ (year)^−1^	Backus & Gross^32^
*w*_*i*_	Genotype specific fitness	Dependent on *s* and *h*	
*μ*	Per-capita migration rate from island to mainland	Varied (10^−4^–1.2 × 10^−1^ (year)^−1^)	
*s*	Drive allele fitness cost	0.8	
*h*	Degree of dominance of fitness cost of drive allele	Non-threshold drive: 0.3 Threshold drive: 0.8	
*e*	Homing probability	0.95	

## Results

### Single patch model

The single-patch behavior of the standard homing drive that we use here has been well-studied previously^2,23,36,37^. Depending on the drive parameters *s*, *h* and *e*, several qualitatively different behaviors can occur following release of drive into an otherwise entirely susceptible population: guaranteed fixation of the drive, guaranteed loss of the drive, co-existence of drive and susceptible alleles at a stable polymorphic equilibrium or invasion threshold behavior (i.e. drive either goes to fixation or is lost, depending on its initial frequency) resulting from the existence of an unstable polymorphic equilibrium. Elimination of the population is possible when the drive remains in the population (going to a stable equilibrium with a positive frequency—either fixation or a polymorphic equilibrium) and imposes a cost that is sufficiently high to bring the reproductive number of the population below one.

We illustrate these dynamics using two sets of parameters: one for which the drive can spread through a susceptible population regardless of its frequency (no invasion threshold scenario), and another for which the drive can only spread when its frequency exceeds an invasion threshold (invasion threshold scenario). In both cases we consider a fitness cost of *s* = 0.8 and a homing probability of *e* = 0.95. The two scenarios differ only in the dominance, *h*, of the drive. For the no invasion threshold scenario we take *h* = 0.3, while for the invasion threshold scenario we take *h* = 0.8, which leads to an invasion threshold frequency of approximately 0.621 (corresponding to a ratio of approximately 1.64:1 drive:wild-type individuals). Releases occur into a population that is at carrying capacity, and population sizes are assessed relative to this carrying capacity.

**Figure 1:**
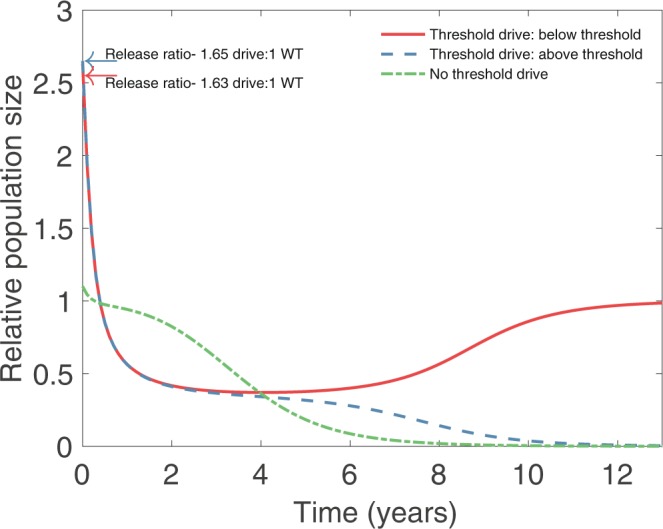
Island population dynamics for the no invasion threshold and invasion threshold scenarios. In both scenarios the drive has an 80% fitness cost (*s* = 0.8) but have differing degrees of dominance: for the no invasion threshold scenario (green curve) we take *h* = 0.3, while for the invasion threshold scenario we take *h* = 0.8. Population sizes are plotted relative to pre-release equilibrium population numbers. In the no invasion threshold scenario, arbitrarily small releases of drive individuals lead to invasion and fixation of the drive allele, leading to suppression of the population (green curve; release of 100 homozygous drive individuals, 0.1:1 release ratio). In the invasion threshold scenario, invasion of the drive depends on whether the initial release exceeds the invasion threshold (for this choice of parameters, the invasion threshold frequency for drive is 0.621, corresponding to approximately 1.64 drive individuals for each wild-type individual). Red curve depicts a sub-threshold release (1630 homozygous drive females; 1.63:1 release ratio), leading to loss of the drive allele and only temporary suppression of the population before its return to carrying capacity. Blue curve depicts a successful release (1650 homozygous drive females; 1.65:1 release ratio), for which the drive invades and reaches fixation, leading to elimination of the population. Values of other parameters are given in Table 1.

Figure 1 shows the population dynamics that result from drive releases in these two scenarios. For the no invasion threshold scenario (green curve), even a small release of drive individuals, so that the initial relative population size is only just above one, leads to spread of the drive allele and hence reduction and eventual elimination of the population. For the invasion threshold scenario (blue and red curves), spread of the drive, and hence the fate of the population, depends on whether the release frequency of the drive is above (blue curve) or below (red curve) the invasion threshold. A sufficiently large release (blue curve) leads to fixation of the drive and elimination of the population, while an insufficient release (red curve) leads to loss of the drive and recovery of the population following a transient period of reduction.

### Island-mainland model

We now turn to the main question of how migration from an island population on which drive individuals have been released will impact a mainland population that has a mix of susceptible and resistant individuals. To present something approaching a worst-case scenario, we first assume that the frequency of resistant individuals on the mainland is rather low, with a resistance allele frequency of 5% and susceptible allele frequency of 95%. We take our island population to be 1/100^th^ the size of the mainland population, with unidirectional migration from the island to the mainland occurring at per-capita rate of 0.012 per year, corresponding to movement at the rate of one mouse per month at baseline.

**Figure 2:**
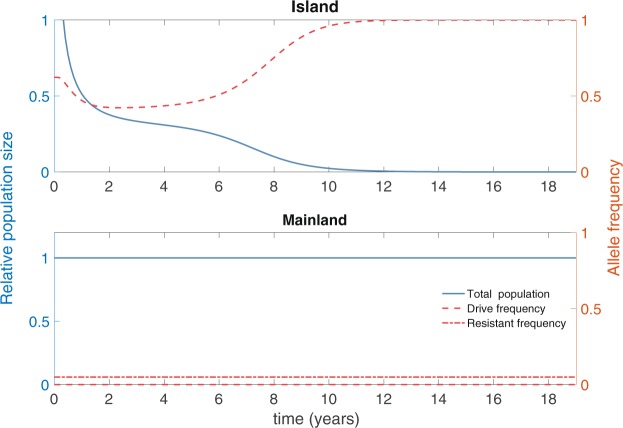
Island and mainland population dynamics (blue curves) and genetics (red curves) in the invasion threshold scenario (*s* = 0.8 and *h* = 0.8), following an above-threshold release of drive individuals on the island (1650 drive homozygotes released at *t* = 0; release ratio 1.65:1). Left axis (blue) denotes population size relative to pre-release equilibrium, right axis (red) denotes allele frequency. The drive spreads on the island (dashed red curve shows allele frequency for drive), suppressing its population (solid blue curve shows relative population size). Migration from the mainland to the island (at baseline, on average one island individual travels to the mainland a month) leads to a low drive frequency on the mainland that does not exceed the invasion threshold there. Consequently, the mainland population is largely unaffected. (Dot-dashed line denotes frequency of resistance allele. Note that the target allele being fixed means that the resistant allele is not present on the island).

For the invasion threshold drive scenario, we consider an island release that is above threshold, so that the drive approaches fixation in the long run (Fig. 2, upper panel, red curve) and the island population is successfully eliminated (Fig. 2, upper panel, blue curve). Even though drive individuals migrate from the island to the mainland, the large size of the mainland population means that the drive frequency remains small and never exceeds the invasion threshold. Thus, the drive cannot spread on the mainland, leaving the size and genetic composition of the mainland population largely unaffected.

**Figure 3:**
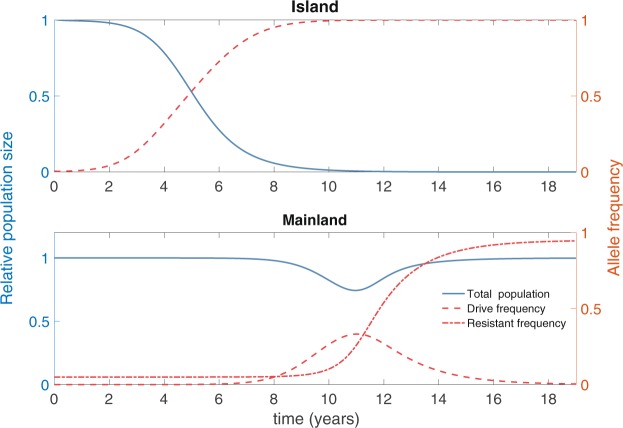
Island and mainland population dynamics and genetics in the no invasion threshold scenario (*s* = 0.8 and *h* = 0.3). As in Fig. 2, blue curves and axes denote population sizes, measured relative to pre-release equilibria, while red curves and axes denote allele frequencies. One hundred homozygous drive individuals (0.1:1 release ratio) are released at time *t* = 0. We assume that resistance is very low on the mainland (allele frequency of just 5%), representing a rather pessimistic scenario in terms of the susceptibility of the mainland population to the drive. The drive spreads to fixation and suppresses the island population. Migration to the mainland (at baseline, on average one island individual travels to the mainland a month) means that the drive is introduced to the mainland, where it can spread through the susceptible population, but not the resistant population. The total population undergoes a temporary suppression as the drive spreads through the susceptible population. The frequency of resistant alleles increases as a result of drive, and density dependent population regulation returns the mainland population to the pre-release equilibrium level.

The situation is different in the no invasion threshold scenario. The drive is able to spread on the mainland even when present at the low frequencies that arise due to migration of drive individuals from the island. Individuals with a resistance allele are unaffected by the drive, however, and so spread only occurs through the susceptible individuals. Again, the drive spreads on the island (Fig. 3, upper panel, red curve) causing successful elimination of the island population (Fig. 3, upper panel, blue curve). On the mainland, drive spreads through the susceptible portion of the population (Fig. 3, lower panel, red dashed curve) causing a reduction in the population size (Fig. 3, lower panel, blue curve) due to fitness costs incurred by drive-bearing individuals. Because they are unaffected by the drive, resistant individuals benefit from having a larger relative fitness in the presence of drive individuals and so their frequency increases (Fig. 3, lower panel, red dot dashed curve), while the drive frequency decreases. As the frequency of resistant individuals increases, the average fitness of the population returns to the level seen initially, and density-dependent dynamics returns the population to its original size. In this setting, we see a transient reduction in the mainland population size before a recovery due to the presence of resistance. The genetic composition of the mainland undergoes a shift during this process, with a reduction in the frequency of susceptible alleles (although not their elimination) and a corresponding increase in the frequency of resistance (see Supplementary Information and Supplementary Fig. 3).

In the Supplementary Information we show that high levels of resistance prevent the spread of drive on the mainland. Using a linear invasion analysis, we show that drive cannot invade when the initial frequency of the resistant allele is above 1 − *hs*/[*e*(1 − *hs*)].

**Figure 4:**
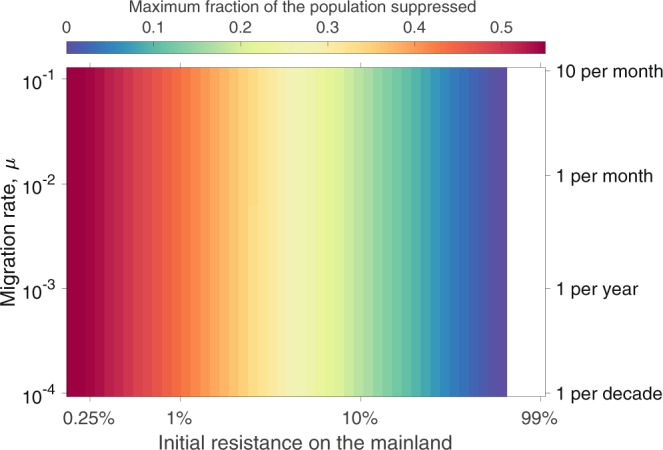
Maximum level of transient suppression seen on the mainland following an island release of 100 homozygous drive individuals in the no invasion threshold scenario (*s* = 0.8 and *h* = 0.3) across combinations of different migration rates and initial frequencies of resistant alleles on the mainland. Different levels of suppression are denoted by different colors (see color key on figure). Low initial resistance frequencies depict pessimistic scenarios (mainland is almost entirely susceptible to the drive), while high initial resistance frequencies approach the private allele scenario discussed in the text. The white region of the figure denotes initial levels of resistance that exceed the threshold level above which drive cannot invade the mainland.

**Figure 5:**
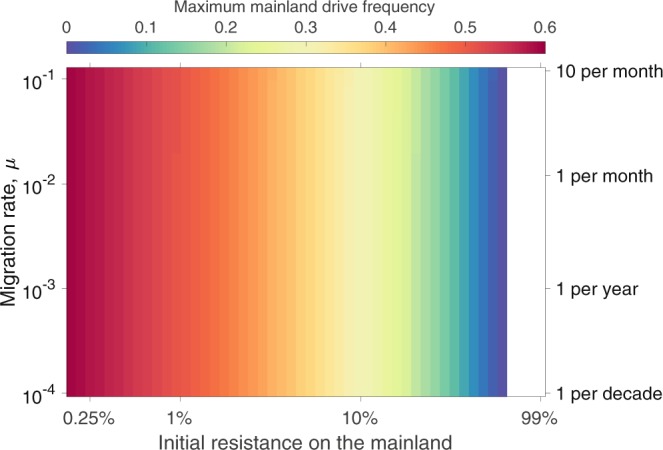
Maximum drive frequency observed on the mainland following an island release. Maximum drive frequency is indicated by color. All other details as in Fig. 4.

Figures 4 and 5 explore how the magnitude of population suppression and the maximum allele frequency of the gene drive observed on the mainland depend on the initial level of resistance on the mainland and the migration rate from the island to the mainland in the no invasion threshold scenario. Naturally, the level of transient suppression (fractional reduction below initial equilibrium) on the mainland depends strongly on the frequency of resistant and susceptible individuals there (Fig. 4), although an important observation is that the level of transient suppression is considerably lower than the initial frequency of susceptible individuals. The level of migration is seen to have little impact on the level of suppression. Similarly, the initial composition of the mainland population impacts the peak drive frequency reached on the mainland, while the level of migration again has little impact (Fig. 5). For the assumed demographic and drive parameters, 10% or higher levels of resistance on the mainland lead to a transient population suppression of at most 20% and peak drive frequency below 30%. Alternative choices for demographic and drive parameters would impact these numbers (see sensitivity analyses in the Supplementary Information). Obviously, in the PA setting, drive would be unable to spread on the mainland, but Figs 4 and 5 reveal that even in situations for which the susceptible allele is not absent, but only fairly uncommon on the mainland, the levels of suppression that would result would be small, and quite likely smaller than natural fluctuations in population size that might result from demographic or environmental stochasticity.

The peak level of suppression and the maximum drive frequency on the mainland occur at roughly similar times. The timing of these events is relatively insensitive to the initial frequency of resistance on the mainland and the migration rate over a wide range of conditions (see Supplementary Information and Supplementary Figs 1 and 2), with dynamics playing out over the course of 10 to 20 years. High initial levels of resistance, however, can lead to much slower spread of the drive on the mainland. As mentioned above, drive cannot invade when the initial frequency of the resistant allele is above 1 − *hs*/[*e*(1 − *hs*)]. Invasion of drive will occur only slowly (and with a low peak level) when the frequency of resistance is not too far below this level. Supplementary Fig. 4 shows that the peak level of suppression on the mainland is almost independent of the size of the release on the island, while the time until this suppression occurs is only weakly dependent on the release size.

## Discussion

There is a fundamental tension between the ability of a gene drive to spread locally within a target area and its ability to invade populations beyond that area. For a non-threshold gene drive designed for suppression or elimination, the potential impact outside the targeted area can be of serious concern even if there is a likelihood that resistance to the drive will evolve before irreversible damage is done. Indeed, such concerns were strongly expressed in many of the seminal early papers on gene drive (e.g.^2,3^). Clearly, mechanisms that give some level of control over the spread of a gene drive are prerequisites for the deployment of a gene drive for suppression or elimination of a targeted population if other populations of the species contribute positively to biodiversity or economics. Utilizing the high degree of genetic specificity exhibited by homing drives, the LFA approach provides one option to limit the impact of unintended spread of a drive. In the fortunate, although perhaps rather unlikely special case of private alleles, where all individuals in non-target populations lack the susceptible allele and the susceptible allele is fixed on the island, the drive would be completely confined to the target population.

The LFA approach is related to two-step gene drive approaches that first spread a target allele into a population and then release a second drive that is specific to the target allele. For example, Esvelt *et al*.^4^ suggested this approach for island populations, arguing that containment would happen provided that release of the second drive occurred before any individuals bearing the first drive escaped the island. The uncertainty involved in this scenario could be problematic for some stakeholders and regulatory authorities. In our approach, naturally occurring targeted alleles are expected to be fixed within small island populations due to genetic drift or founder effects that have occurred in the past. These targeted alleles may be present in other locations, but as long as they are not fixed, impacts on populations in those locations should be transient.

As demonstrated by other mathematical models (e.g.^22,38^) some spatially limited gene drive mechanisms are unlikely to function well for strongly suppressing populations, because these require extremely large releases, which are neither feasible nor advisable for pest species on islands (but see^39^ for an alternative approach for localized suppression of mainland populations). In this regard, the LFA approach stands out for its predicted ability to efficiently eliminate a small mostly isolated population without impacting other populations beyond a transient effect. The LFA approach could also be useful in the setting of a suppression or replacement drive that does not lead to elimination of the island population, although, as discussed in detail in the Supplementary Information (Section S.5), persistence of the drive on the island will mean continual reintroductions of drive to the mainland via migration, most likely leading to maintenance of drive on the mainland at a low frequency.

Our analysis employed a deterministic model, which allows for continuous migration from island to mainland. This model describes the average behavior of the system and so gives a good indication of expected dynamics in a large well-mixed mainland population. It describes the magnitude of suppression and its timing, but does not account for the discrete numbers of individuals in populations. Given that migration from the island to mainland is anticipated to occur as relatively infrequent events that involve small numbers of individuals, a stochastic model would be more realistic. Such a model would allow questions about the likelihood of the occurrence of migration events and of resulting transient spread of the drive, and distributions of the magnitude and timing of any transient suppression that results. However, we would not expect the qualitative results of such a model to predict a qualitatively different outcome from the current model in terms of impacts on the overall mainland population (see Supplementary Information for results from a stochastic model and some additional discussion of the impact of stochasticity).

For this proof-of-concept study of the LFA approach, we employed a highly simplified description of the population dynamics and genetics of the system. Many ecological and behavioral complexities, such as Allee effects^40^, the spatial and social structures of mouse populations, and mouse mating behavior, will impact the dynamics of population suppression and elimination under the action of gene drives. More refined models that include such features will have to be developed if the use of this approach is to be considered for a real-world release program.

It is important to recognize that one of the outcomes of mice arriving on the mainland that have a homing drive with no threshold is that the frequency of the resistant allele will increase. If after the mice are eliminated from the island a mouse or a few mice carrying the now more common resistant allele migrate to the island, it is likely that the new population on the island would no longer be a good target for the previously used construct. However, if recolonization was started by one or a few individuals, founder effects and drift would be likely to result in other fixed alleles that could be targeted.

There are a number of technical and societal challenges in moving the LFA from concept to application. While there is empirical evidence of lower polymorphism in small island populations (e.g.^41–43^), not every fixed allele in an island population will be a good target for a CRISPR-based gene drive. Appropriate targets will depend on the intended nature of the drive (e.g. if it is to be sex-specific, employ a split-drive design, etc), impacting the number of available loci. Furthermore, these alleles will optimally have at least two sites that can be targeted by the CRISPR-CAS nuclease complex to decrease the chance of drive failure due to resistant alleles arising through non-homologous end joining^44^. It will be critical that full genomes of individuals be scanned for the best possible targets and those targets will need to be scrutinized for potential problems. Also, because the LFA approach requires that the target allele is fixed, island populations will need to be sampled and evaluated extensively before moving ahead with any genetic engineering to ensure an acceptably high probability that the targeted allele is truly fixed on the island. This will require focused sequencing of the target loci in a large number of individuals, but it should be recognized that 100% assurance is not possible.

At a societal level, lack of an ecological impact of an LFA in a non-target location of the specific target species must be assured. Models that include details of the species biology, population structure and genetics will be critical in moving toward regulatory approval. But assurance of lack of an ecological impact of LFA constructs on the non-targeted populations may not be sufficient for some stakeholders who will have a concern with even one engineered individual arriving in their area.

Beyond direct impacts of an LFA, researchers must be cognizant of the fact that in developing the technology for LFA in a problematic species, they are also developing tools that could be used by others to construct unrestricted gene drives in that species. There will always be tradeoffs in developing new technologies and there are unlikely to be simple decisions. Vigilance and input from diverse stakeholders “early and often”^45^ will be critical in coming to decisions.

## Supplementary information

Supplementary Information

Dataset 1

Dataset 2
