# Supplementary material for: Locally Fixed Alleles: A method to localize gene drive to island populations
Source: Sci Rep. 2019 Nov 1;9:15821. doi: 10.1038/s41598-019-51994-0 (PMC6825234; doi:10.1038/s41598-019-51994-0)
Supplement: Supplementary file 1 — Supplementary Information [file 41598_2019_51994_MOESM1_ESM.pdf]

Supplementary Information for

Locally Fixed Alleles: A method to localize gene drive to island populations

Jaye Sudweeks [a], Brandon Hollingsworth [b], Dimitri V. Blondel [c], Karl J. Campbell [d], Sumit Dhole [e], John D. Eisemann [f], Owain Edwards [g], John Godwin [c,h], Gregg R. Howald [d], Kevin P. Oh [f,i], Antoinette J. Piaggio [f], Thomas A. A. Prowse [j], Joshua V. Ross [j], J. Royden Saah [d,h], Aaron B. Shiels [f], Paul Q. Thomas [k], David W. Threadgill [l], Michael R. Vella [b], Fred Gould [e,h] and Alun L. Lloyd [a,b,*]

(a) Department of Mathematics, North Carolina State University, Raleigh, NC 27695, USA
(b) Biomathematics Graduate Program, North Carolina State University, Raleigh, NC 27695, USA
(c) Department of Biological Sciences, North Carolina State University, Raleigh, NC 27695, USA
(d) Island Conservation, 2100 Delaware Ave., Suite 1, Santa Cruz, CA 95060 USA
(e) Department of Entomology and Plant Pathology, North Carolina State University, Raleigh, NC 27695, USA
(f) National Wildlife Research Center, US Department of Agriculture, Fort Collins, CO 80521, USA
(g) CSIRO Land & Water, Centre for Environment and Life Sciences, Floreat, WA, Australia
(h) Genetic Engineering and Society Center, North Carolina State University, Raleigh, NC 27695, USA
(i) Department of Microbiology, Immunology & Pathology, Colorado State University, Fort Collins, CO 80523, USA
(j) School of Mathematical Sciences, The University of Adelaide, Adelaide, SA 5005, Australia
(k) The Robinson Research Institute and School of Medicine, The University of Adelaide, Adelaide, SA 5005, Australia
(l) Department of Molecular and Cellular Medicine, Texas A&M University, College Station, TX 77843, USA

* Corresponding author, email: alun_lloyd@ncsu.edu

Contents:

S.1. Threshold for Invasion of Drive into Partially Susceptible/Partially Resistant Mainland Population

S.2. Additional Details of Dynamics on the Mainland: Timing of Suppression and Peak Drive Level; Long-Term Dynamics

S.3. Sensitivity of Results to Parameter Values

S.3.1. Sensitivity to Drive Parameters

S.3.2. Sensitivity to Demographic and Density Dependence Parameters

S.3.2.1 Logistic Model

S.3.2.2. Generalized Logistic Model

S.4. Stochastic Model

S.5. Population Suppression and Population Replacement

S.1. Threshold for Invasion of Drive into Partially Susceptible/Partially Resistant Mainland Population

It can be shown that there is a threshold level of resistance above which drive cannot invade the mainland. This threshold can be derived by examining the stability of a drive-free equilibrium state of the single patch model, using the standard approach of linearizing the model about the equilibrium (see, for example[1]). This process involves calculation of the eigenvalues of the Jacobian matrix (i.e. the matrix of the partial derivatives of the right-hand sides of the differential equations (eqns 3 in the main text) with respect to the state variables of the model) evaluated at the equilibrium of interest. The 6x6 Jacobian matrix and its eigenvalues are easily calculated with the assistance of a computer algebra package (e.g. Maple [Maplesoft, a division of Waterloo Maple, Inc., Waterloo, Ontario] or Mathematica [Wolfram Research, Inc., Champaign, IL]). A Maple worksheet providing details of the calculations is provided as a supplementary file.

Given that we assume equal fitness of susceptible and resistant types, in the absence of drive there is no selection between susceptible and resistant types and so we have non-unique drive-free equilibria:

$$N_{\mathrm{SS}} = (1-q_R)^2 N^*,\ N_{\mathrm{SR}} = 2q_R(1-q_R)N^*,\ N_{\mathrm{RR}} = q_R^2 N^*,\ N_{\mathrm{SD}} = N_{\mathrm{RD}} = N_{\mathrm{DD}} = 0$$

Here q_{R} is the frequency of the resistant allele and N^* is the wild-type (i.e. drive-free) equilibrium population size (equal to $(\lambda-\rho)/(\lambda q+\alpha)$) . We note that these equilibria form a curve in phase space.

The linear stability analysis gives six eigenvalues, one of which is zero (corresponding to neutral stability along the curve of drive-free equilibria), one equal to $\rho-\lambda$ and three equal to $-\lambda(q\rho+\alpha)/(\lambda q+\alpha)$. We see that these last four eigenvalues are negative (note that λ must be greater than ρ in order for N^* to be positive). The ability of drive to invade (from low initial frequency) is determined by the sign of the final eigenvalue, whose value is equal to $\lambda[(1-q_{\mathrm{R}})(1-hs)e-hs](q\rho+\alpha)/(\lambda q+\alpha)$. Invasion is only possible if this quantity is positive, and so consideration of the quantity in square brackets leads to the condition

$$q_R < 1 - hs/\{e(1-hs)\}.$$

Another way to derive this threshold (and one that immediately applies to a number of previous studies in the literature) is by considering a discrete-time (discrete generations) description framed in terms of allele frequencies (see, for example[2,3]).

If the allele frequencies for Susceptible, Drive and Resistant alleles in the current generation are written as q_{S}, q_{D} and q_{R}, it can be shown that the frequency of the drive allele in the next generation, q_D', will be given by

$$q_D' = \frac{1}{\overline{w}}\left(\{1-s\}q_D^2 + \{1-hs\}q_S\, q_D\,\{1+e\} + \{1-hs\}q_R\, q_D\right),$$

where $\overline{w}$ is the mean fitness, which equals

$$\overline{w} = 1 - sq_D^2 - 2hsq_D\left(q_S + q_R\right).$$

When thinking about invasion of drive into a population that initially consists of susceptible and resistant alleles, q_D will be small, and so the following linear equation can be derived for the change in the frequency of the drive allele from one generation to the next:

$$q_D' - q_D \approx q_D\left(\{1-hs\}q_S\,\{1+e\} + \{1-hs\}q_R - 1\right).$$

(This equation is correct to first order in q_D .)

We see that the drive frequency can only increase if the term in parentheses is positive, meaning that

$q_R < \frac{e(1-hs)-hs}{e(1-hs)}$,

which may be written as

$$q_R < 1 - hs/\{e(1-hs)\}.$$

S.2. Additional Details of Dynamics on the Mainland: Timing of Suppression and Peak Drive Level; Long-Term Dynamics

Supplemental Figures 1 and 2 explore the timing of (1) suppression on the mainland and (2) the peak level of drive seen on the mainland. These two events occur at similar times (but not at exactly the same time). We note that over a wide range of initial levels of resistance and migration rates, there is only weak dependence of either time on these quantities, and that these times increase substantially as the initial level of resistance approaches the non-invasion threshold.

Supplemental Figure 3 explores the composition of the mainland population that remains after the transient spread of drive, showing the long-term (100 year) frequency of the susceptible allele. We note that, while the transient spread of drive leads to the reduction of susceptible alleles on the mainland, it does not lead to their elimination.

Supplemental Figure 4 shows that the peak level of suppression and the maximum drive frequency on the mainland are almost independent of the size of the release on the island (that they are not constant cannot be seen at the scale shown on this figure: both curves exhibit a very weak dependence on release size). The time until the peak suppression occurs is only weakly dependent on the release size for biologically plausible release sizes, varying only by about 3 years for release sizes between 1 and 1000 individuals, but continuing to increase in the limit as the release size approaches zero. In the deterministic model, invasion is possible from arbitrarily small releases, although it takes increasingly long for drive to increase to appreciable levels from extremely small release levels, hence the time to minimum continues to increase. Note that the model behaves qualitatively differently when the release size is zero from when the release size is non-zero.

Supplemental Figure 1. Dependence of the time until the mainland population achieves its minimum on the initial level of resistance on the mainland and the migration rate from the island. All other details are as in Figure 4 of the main text. The white region of the figure denotes initial levels of resistance that exceed the threshold level above which drive cannot invade the mainland.

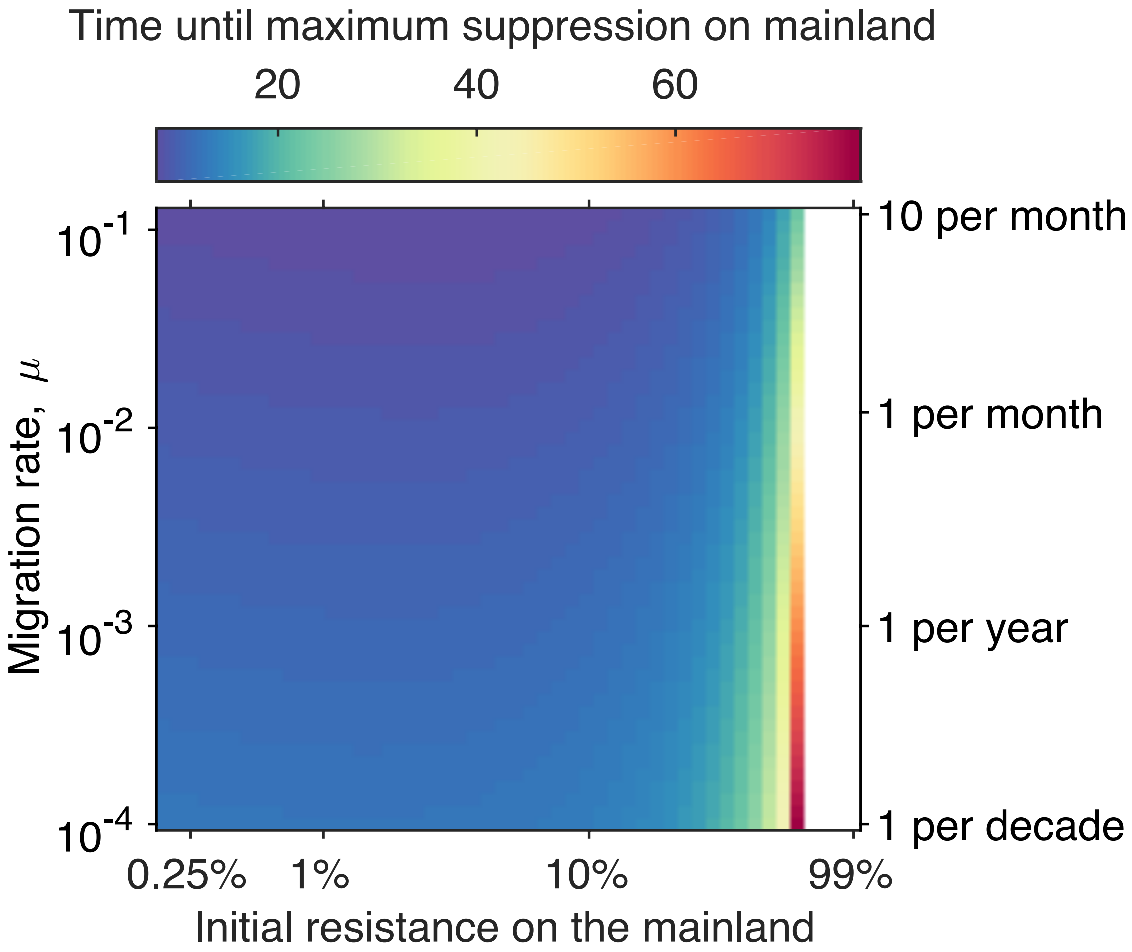

Supplemental Figure 2. Dependence of the time until the drive frequency achieves its maximum on the mainland on the initial level of resistance on the mainland and the migration rate from the island. All other details are as in Figure 4 of the main text.

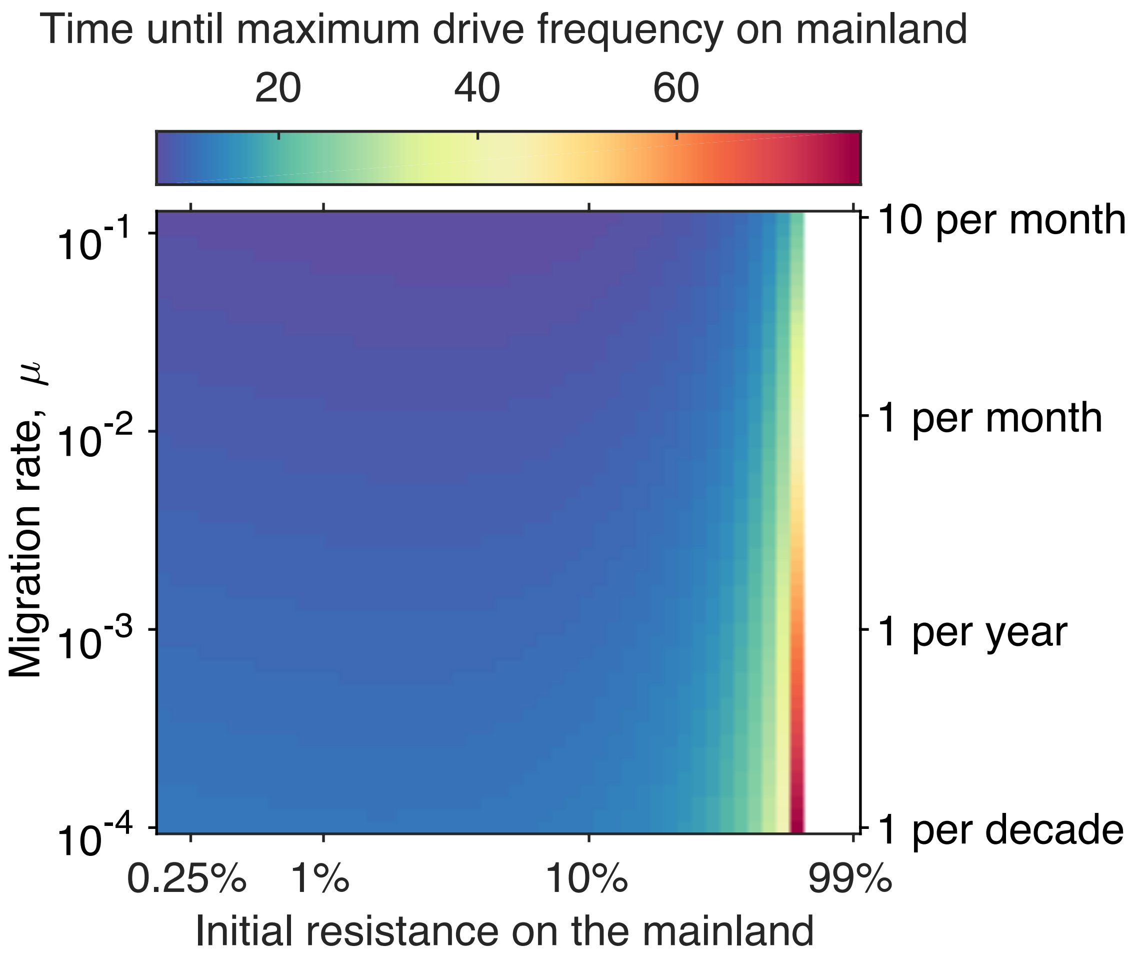

Supplemental Figure 3. Frequency of the susceptible allele on the mainland 100 years after an island release in the no invasion threshold scenario (*s* = 0.8 and *h* = 0.3) across combinations of different migration rates and initial frequencies of resistant alleles on the mainland. All other details are as in Figure 4 of the main text. Note that susceptible alleles remain in the population after the transient spread and loss of drive on the mainland. The white region of the figure denotes initial levels of resistance that exceed the threshold level above which drive cannot invade the mainland.

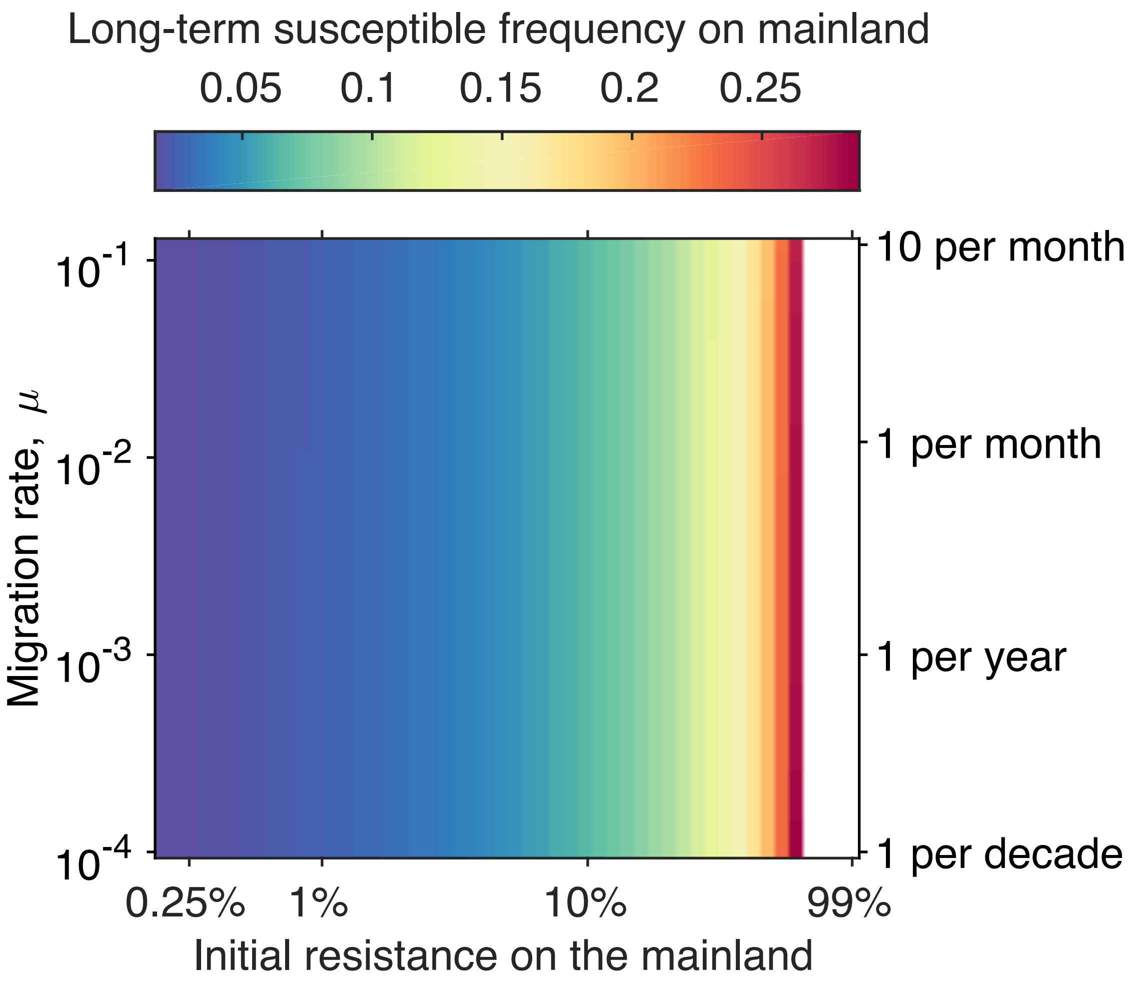

Supplemental Figure 4. (Both panels) Maximum level of transient suppression (dashed blue curve; read scale on left axis), time taken for this level of suppression to occur (red curve, read scale on right axis) and maximum level of gene drive (solid blue curve; read scale on left axis) seen on mainland following releases of various sizes on the island under the no invasion threshold scenario. Initial frequency of resistance on the mainland is equal to 0.05 and migration occurs at a per-capita rate of 0.012 per year. All other parameters are as in Figure 3 of the main text. Circles on the vertical axis denote that the curves are not continuous when the release size is zero: the behavior of the model is qualitatively different between zero and positive release sizes. The bottom panel depicts the same results but using a logarithmic scale on the horizontal axis to emphasize the mathematical behavior as the release size approaches zero.

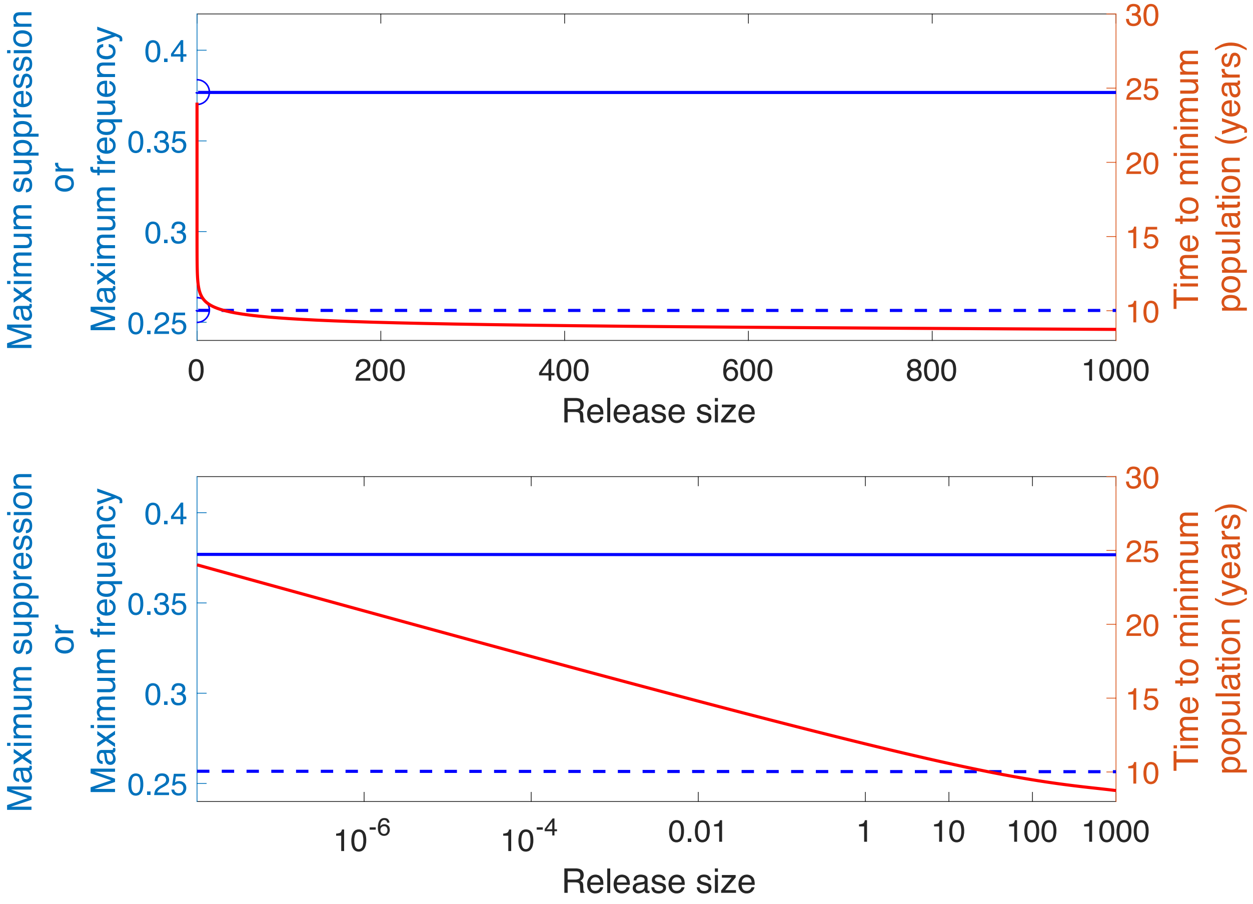

S.3. Sensitivity of Results to Parameter Values

In this section we explore how model results, specifically the maximum level of suppression seen on the mainland and the peak level reached by drive on the mainland, depend on the drive and ecological parameters used. Such a sensitivity analysis provides increased confidence in the utility of the LFA approach.

Before exploring the impact of individual parameters in detail, we performed a global sensitivity analysis involving four parameters: the fitness cost of the drive, *s*, the dominance of this fitness cost, *h*, the homing probability, *e*, and the demographic parameter λ, the female fecundity parameter (i.e. the female per-capita birth rate of the population at low population densities, when density-dependent reductions in birth rate can be ignored). As discussed below (S.3.2.1), for the logistic model, if we choose to fix the equilibrium population size, there is only one demographic parameter, the net per-capita birth/death rate at low population densities, that can be independently varied. We choose to do this by varying the per-capita birth rate (female fecundity, λ).

Given that we have little to no information on the uncertainties of these parameters about their baselines, we employed uniform distributions for their possible values (Table S.1), following the approach taken by Prowse et al.[4] in their sensitivity analysis of the Y-CHOPE Y chromosome shredding gene drive. We then used a sampling-based variation decomposition approach[5] (Fourier Analysis Sensitivity Test, FAST, implemented using the SAFE Toolbox[6]) to assess the contributions of the uncertainties in different parameters to the variation seen in model outputs across the parameter space. This approach is somewhat akin to more familiar analysis of variance statistical methods. 5000 simulation runs were carried out, each using a set of parameters sampled from the parameter space described in Table S.1.

Supplemental Table 1: Baseline values and assumed distributions for parametric sensitivity analysis.

Parameter	Baseline	Distribution
s	0.8	U(0.65,0.95)
h	0.3	U(0.1,0.5)
e	0.95	U(0.7,1.0)
λ	8.4	U(6,10)

Across the simulation runs based on 5000 sets of parameters, the range of observed maximum suppression values fall between less than 1% and 40.4%, with a mean of 20.8% and standard deviation of 0.08% (coefficient of variation of 38.8%). For the maximum drive frequency, the range of observed values was between less than 1% and 53.7%, with a mean of 31.8% and standard deviation of 12.2% (coefficient of variation of 38.2%).

For maximum suppression observed on the mainland, the first order effects of the four parameters explained 84% of the variation. Two of the drive parameters, *h* and *e*, and the demographic parameter λ explained fairly similar amounts of variation (26%, 22%, and 23%, respectively), while the fitness cost *s* explained just 12% of the variation. This analysis says that if we wished to predict the impact of LFA on

mainland population suppression, reducing the uncertainty in either *h*, *e*, or λ has a bigger impact on the confidence in our predictions than reducing uncertainty in *s*.

For maximum drive frequency observed on the mainland, the first order effects of the four parameters explained 94% of the variation. The drive fitness parameters *s* and *h* have the biggest impact, explaining 47% and 38% of variation, respectively. The homing probability *h* has a much smaller impact, explaining 8% of variation, while the demographic parameter λ has very little impact on the maximum drive frequency, explaining less than 0.1% of variation (see S.3.2.1 for more discussion on this).

S.3.1. Sensitivity to Drive Parameters

Figure S.5 shows the dependence of the maximum levels of suppression and drive seen on the mainland on the drive fitness cost and dominance of this fitness cost. Results are shown for the region of drive parameter space for which the island population is eliminated and for which no threshold behavior is observed (Deredec et al.[2] and Alphey and Bonsall[7] provide analytic expressions for the locations of these boundaries). The dashed line on the plot shows the boundary between the parameter regions for which drive becomes fixed or approaches a polymorphic equilibrium with wild-type[2,7].

Figure S.6 shows sensitivity of outcomes over a region of drive parameter space assuming different values for the homing probability, and Figure S.7 shows dependence of outcomes on the initial level of resistance on the mainland assuming different values for the homing probability.

Supplemental Figure 5: Heatmaps showing the dependence of (panel a) the maximum suppression observed on the mainland and (panel b) the maximum level of drive seen on the mainland on the drive parameters *s* and *h*. The scales on the color bars are chosen to be the same as in Figures 4 and 5. White regions on this figure denote combinations of drive parameters that either lead to threshold behavior, loss of drive or for which the drive fails to lead to elimination of the island population. The initial frequency of the resistance allele on the mainland is 95%, and the migration rate is 0.012/year. All other parameters are as in Figures 4 and 5 of the main text. The black asterisk denotes the drive parameters used in Figures 4 and 5 of the main text. The dashed line denotes the boundary between the region of parameter space where drive approaches fixation (to the left of the line) and where drive approaches a polymorphic equilibrium with wild-type (to the right of the line).

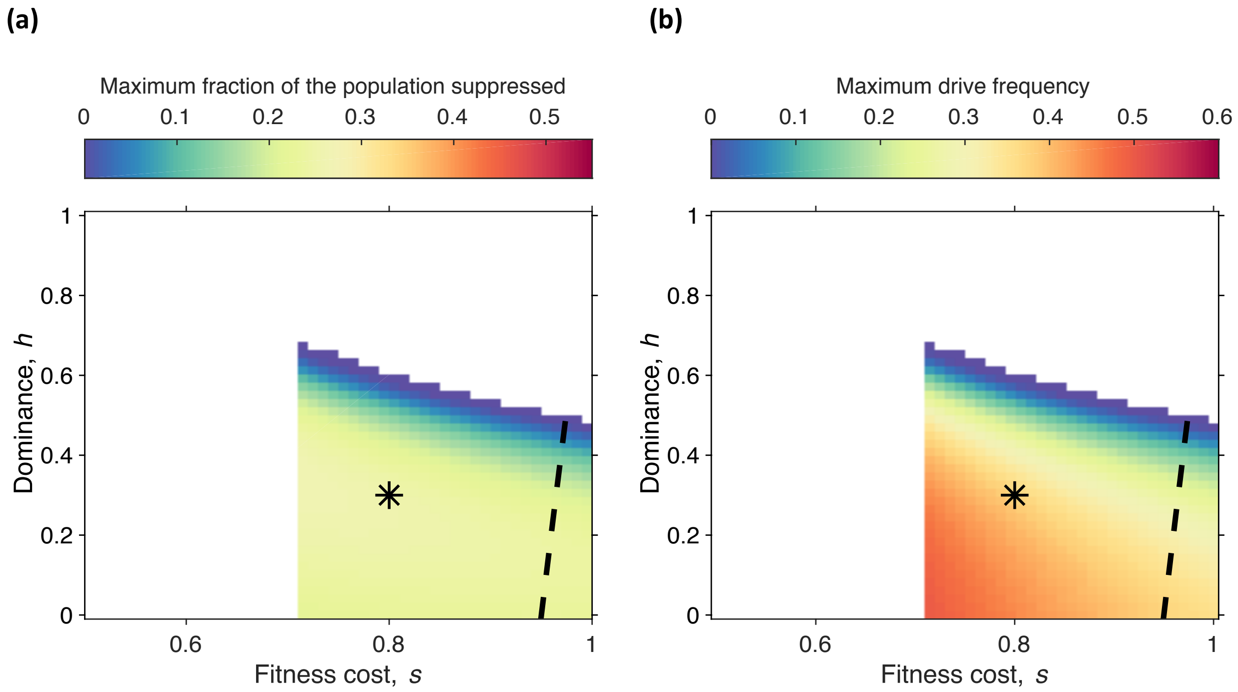

Supplemental Figure 6: Heatmaps showing the dependence of (panels a and c) the maximum suppression observed on the mainland and (panels b and d) the maximum level of drive seen on the mainland on the drive parameters *s* and *h*, and for homing probabilities of (panels a and b) 0.8 and (panels c and d) 0.5. The scales on the color bars are chosen to be the same as in Figures 4 and 5 of the main text. White regions on this figure denote combinations of drive parameters that either lead to threshold behavior, loss of drive or for which the drive fails to lead to elimination of the island population. The initial frequency of the resistance allele on the mainland is 95%, and the migration rate is 0.012/year. All other parameters are as in Figures 4 and 5 of the main text. The black asterisk denotes the drive parameters used in Figures 4 and 5 of the main text. The dashed curve denotes the boundary between the region of parameter space where drive approaches fixation (to the left of the curve) and where drive approaches a polymorphic equilibrium with wild-type (to the right of the curve).

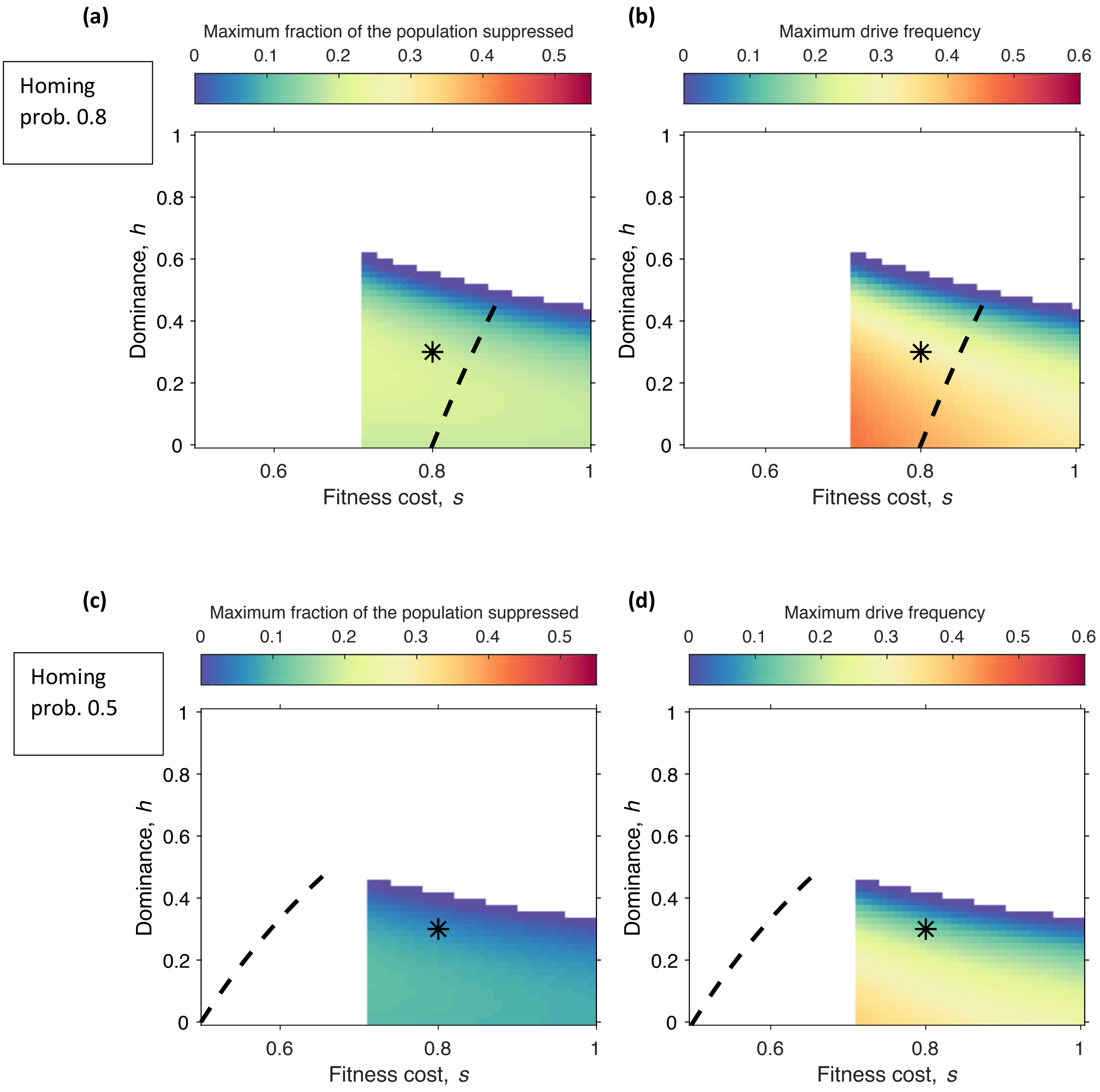

Supplemental Figure 7. (Panel a) Maximum population suppression seen on the mainland and (Panel b) maximum drive frequency seen on mainland for various levels of mainland initial resistant allele frequency, and for different values of the homing probability. In all cases, drive parameters are taken to equal *s*=0.8 and *h*=0.3. All other parameters are as in Figure 3. of the main text.

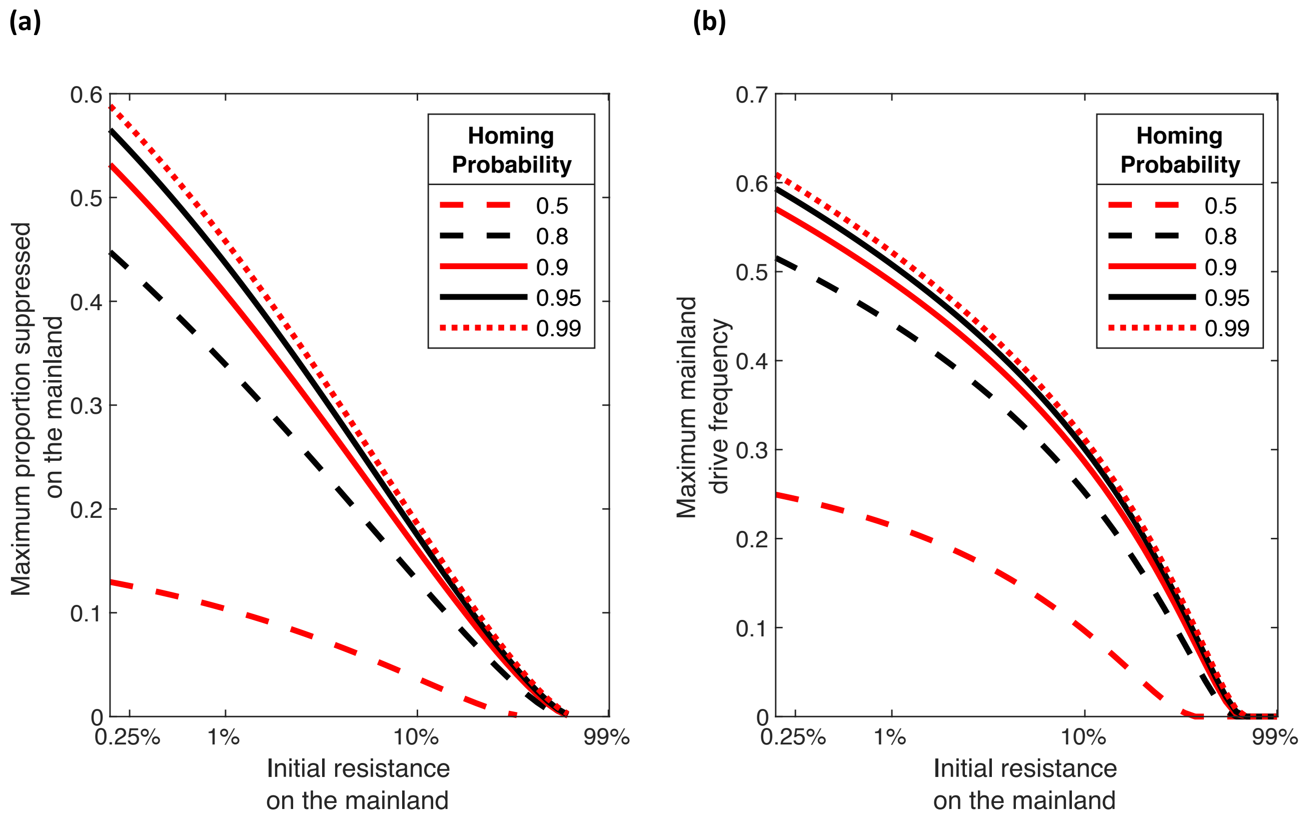

S.3.2. Sensitivity to Demographic and Density Dependence Parameters

S.3.2.1 Logistic Model

We also explore the dependence of our results to demographic and density dependence parameters. The dynamics of the deterministic logistic model involves two parameter combinations: the per-capita growth rate of the population at low densities ($\lambda-\rho$) and the per-capita rate of change of the population growth rate. In order to make comparisons across parameter values, we choose to keep the equilibrium population size fixed. This leaves us with the single parameter combination $\lambda-\rho$ that can be varied in order to explore the impact of demographic parameters within this demographic model. Here, we choose to vary λ in order to achieve this. The primary impact of varying parameters in this way is to change the stability of the positive equilibrium of the logistic model. A natural way to characterize this is by calculating the *return time* of the equilibrium, a measure of the time taken for perturbations of the population about its equilibrium to decay that is commonly used in the theoretical ecology literature[8]. More precisely, the return time is calculated as the reciprocal of the absolute value of the largest eigenvalue of the Jacobian matrix of the model at its positive equilibrium, $1/|f'(N^*)|$, which for this model simply equals $1/(\lambda-\rho)$. Longer return times mean that the population responds more slowly to perturbations away from equilibrium (the equilibrium is "less stable" in this sense).

Figure S.8(a) shows how the maximum suppression seen on the mainland varies as λ is changed, and Figure S.8(b) reinterprets these results in terms of the resulting return time to equilibrium. We see that the maximum suppression seen on the mainland varies in an intuitive fashion as λ is changed. Longer equilibrium return times lead to higher levels of suppression: longer return times mean that the population responds more slowly to perturbations in its size and thus the fitness cost imposed by drive can push the population down to lower levels. The variation in the maximum suppression as λ changes can also be expressed in terms of the elasticity[9], the ratio between the percentage change in maximum suppression and the percentage change in λ, calculated at the baseline parameter set and assuming that the percentage change in λ is small. This elasticity is equal to -1.22, which means that an X% change in λ leads to an approximate change of -1.22X% in the maximum suppression.

Figure S.8(c) shows that the maximum mainland drive frequency depends only weakly on the demographic parameter λ. This is not surprising: at the level of a single patch, population genetics and ecological dynamics are uncoupled (see[7], for example). The weak dependence seen in the figure reflects the impact of migration between two populations whose sizes are varying. Changing the level of density dependence on the island leads to changing the timing of population reduction on the island, hence changing the numbers of drive individuals moving from the island to mainland over time. Similarly, changing the level of density dependence on the mainland changes the timing of population reduction on the mainland. This, in turn, changes the impact of arriving drive-bearing individuals: how they alter mainland drive frequency depends on the relative numbers of arriving and mainland individuals.

Supplemental Figure 8: Dependence of: (panel a) the maximum suppression observed on the mainland, and (panel c) the maximum drive frequency seen on the mainland as the intrinsic per-capita growth rate of the population (λ) is varied. Panel (b) reinterprets the results of panel (a) in terms of the return time to equilibrium (see text for more details). All other parameters are kept fixed at the baseline values used in Figure 3, except for the parameters *q* that describe the linear decline in the per-capita birth rate with increasing population size. The *q* parameters are varied so as to keep island and mainland population sizes fixed as λ is changed. The red dot on each panel corresponds to the baseline set of demographic parameters used in Figure 3 of the main text.

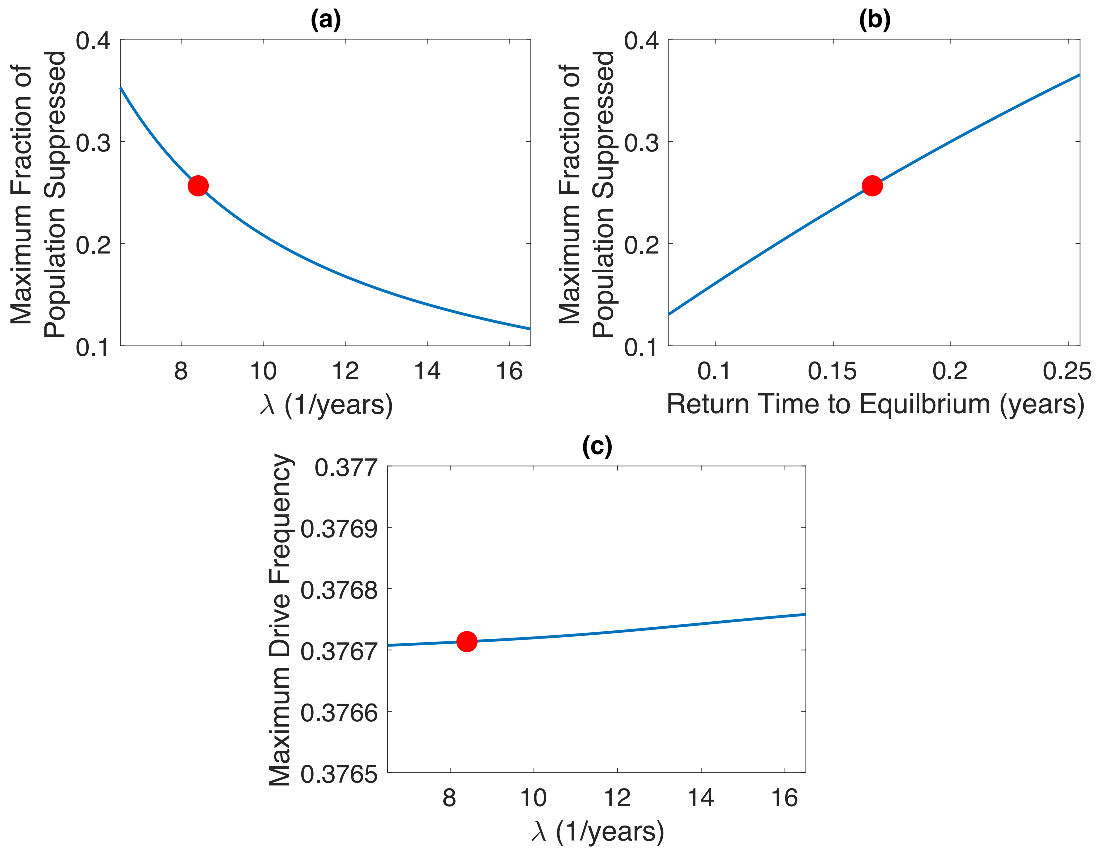

S.3.2.2. Generalized Logistic Model

In the previous section, the sensitivity of model results to the demographic assumptions of the logistic model was explored. As discussed, fixing the equilibrium population size leaves us with relatively little ability to impact demography within the confines of the logistic model framework. We can make a more general exploration of the impact of density dependence by employing a slightly more general population dynamics framework, the generalized logistic model[10,11]. Here we assume that the linearly increasing per-capita death rate of the logistic model is replaced by a nonlinear term that involves the exponent β-1 :

$$\frac{dN}{dt} = \lambda N(1 - qN) - \rho N - \alpha N^{\beta}$$

A β value of 2 corresponds to the logistic model employed in the main text and by Backus & Gross[12]. Values of β above 2 correspond to stronger density dependence, values below 2 to weaker density dependence.

As before, we keep the equilibrium population size constant when making comparisons. There are various ways to do this while varying β, but we employ the simplest choice: we keep the parameters λ, q and ρ fixed, and choose an appropriate value of α. To further simplify our exploration here, we only employ the generalized logistic model on the mainland, maintaining the baseline logistic dynamics on the island. (This means that the timeseries of numbers of migrants arriving on the mainland is kept the same as we vary β, and we explore how changing density dependence on the mainland alters the impact of these migrants on the mainland population.) Furthermore, we assume that density dependence only occurs in the death process on the mainland, i.e. we set the mainland value of q equal to zero.

As expected higher levels of suppression are seen for weaker density dependence ($\beta<2$) and lower levels for stronger density dependence ($\beta>2$), compared to the logistic model ($\beta=2$). The elasticity, calculated at the baseline level of the parameter, is -1.22.

Supplemental Figure 9. Dependence of: (panel a) the maximum suppression observed on the mainland, and (panel c) the maximum drive frequency seen on the mainland as the parameter β that determines the strength of density dependence is varied. Panel (b) reinterprets the results of panel (a) in terms of the return time to equilibrium (see text for more details). It is assumed that density dependence on the mainland only occurs via deaths (i.e. the mainland *q* parameter is set equal to zero). All other parameters are kept fixed at the baseline values used in Figure 3 of the main text, except for the coefficient α of the density dependent (nonlinear) mortality term on the mainland. This parameter is varied so as to keep the baseline (pre-release equilibrium) mainland population size fixed as β is changed. The red dot on each panel corresponds to the baseline set of demographic parameters used in Figure 3 of the main text.

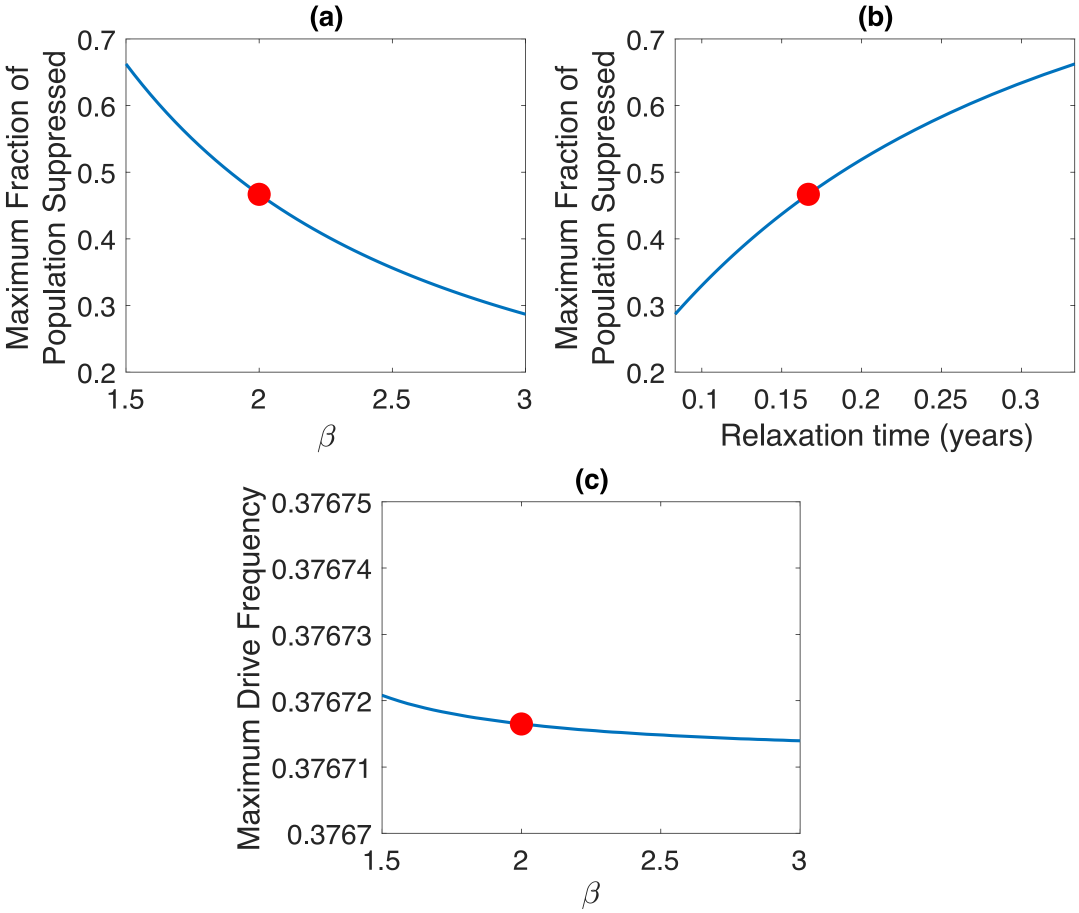

S.4. Stochastic Model

We formulate a stochastic model in the familiar way by reinterpreting the birth, death and migration rates of the deterministic model as rates at which discrete transitions occur in a continuous-time, discrete state Markov chain model (see, for example, Renshaw[13]). Numbers of individuals of each genotype are now integer-valued quantities and processes in the model occur discretely: for instance, migration events involve the movement of a single individual from the island to the mainland. Standard stochastic simulation methods can be used to produce a collection of realizations (simulation runs) of the model.

Because the model is stochastic, repeated simulation starting from the same initial condition leads to variation in observed dynamics. One way to summarize this variability is by a histogram that depicts the distribution of model outcomes across a collection of simulation runs.

An important difference between stochastic and deterministic models is that invasion of drive is no longer guaranteed in the stochastic model, even for choices of parameters and initial conditions for which invasion is certain in the deterministic model. For instance, just by chance it could happen that a drive individual that arrives on the mainland dies before having any offspring there. In general, branching process theory can be used to calculate the probabilities that the arrival of a single drive individual will lead to successful invasion of drive or the failure of drive to spread[14]. These probabilities naturally depend on a number of drive-related parameters. Furthermore, repeated introduction of drive is more likely to lead to successful establishment of drive than a single introduction. For the baseline drive parameters used in the main text (and in this Appendix), numerical simulation shows that the probability of successful spread of drive following the arrival of a single drive individual is approximately 0.315.

For parameters corresponding to Figure 3. in the main text, with a mainland population of N = 100,000, a per-capita migration rate of 0.012/year and the rather pessimistic assumption that the frequency of the resistant allele on the mainland is only 5% (target allele frequency of 95%), we see that the stochastic model gives results that correspond closely to those obtained from the deterministic model. We see a relatively small variation in both the maximum level of suppression and maximum drive frequency seen on the mainland about the values predicted by the deterministic model (Figure S.10).

For a lower level of migration, μ=0.0012/year, we see (Figure S.11) that drive fails to invade on the mainland in a large number of realizations (5,356 out of 10,000). This occurs because at this level of migration, no drive individuals migrated to the mainland before extinction happened on the island in about 14% of the realizations (1,440 out of 10,000). Even if drive-bearing individuals arrived on the mainland, invasion was not guaranteed to occur: drive failed to invade in 3,916 out of the 8,560 realizations in which drive arrived on the mainland (note that some realizations involved two or more arriving migrants). Neither of these two phenomena are captured by the deterministic model, in which migration is a continually-occurring process (minute fractions of individuals continually move from island to mainland) and in which drive can invade from arbitrarily low levels (so the arrival of a fraction of a drive-bearing individual will lead to invasion of drive). Consequently, the deterministic model is in one sense overly pessimistic about the impact of drive on the mainland, in that it predicts that drive is guaranteed to (transiently) invade the mainland. On the other hand, variation about the average behavior in the stochastic model means that the deterministic model can underestimate the impact of

drive when it does invade, although we see that this variation is not so large when the mainland population is large. We note that for the realizations in which drive fails to invade, we do see a non-zero maximum suppression: this reflects the variation that a stochastic wild-type population exhibits about its carrying capacity. (Note that these values would be larger if we observed the population over a longer time interval.)

Supplemental Figure 10. Histograms showing (a) maximum suppression seen on the mainland and (b) maximum frequency reached by drive on the mainland across 10,000 realizations of the stochastic model. Parameter values are as in Figure 3. of the main text, with a per-capita migration rate of 0.012/year and a mainland population size of 100,000.

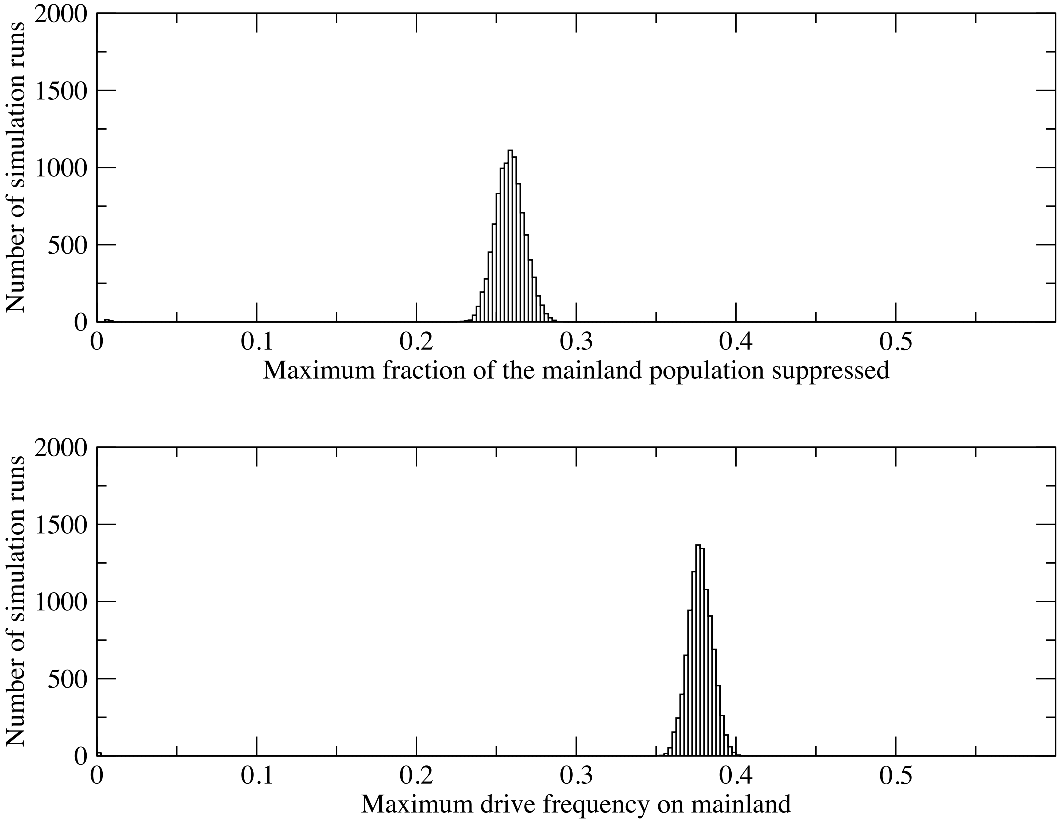

Supplemental Figure 11. Histograms showing (a) maximum suppression seen on the mainland and (b) maximum frequency reached by drive on the mainland across 10,000 realizations of the stochastic model. Parameter values are as in Figure S.10, except that the per-capita migration rate is now lower, at 0.0012/year. Note that the observed results now exhibit bimodality: there are now a substantial number of simulation runs for which the maximum drive frequency is 0 (or low) and the maximum suppression is low. Note that the choice of scale on the vertical axis (chosen to allow the upper part of the bimodal distributions to be clearly visualized) truncates the lower parts of the bimodal distributions. 5,356 out of 10,000 simulation runs fall into the lower parts of the two distributions.

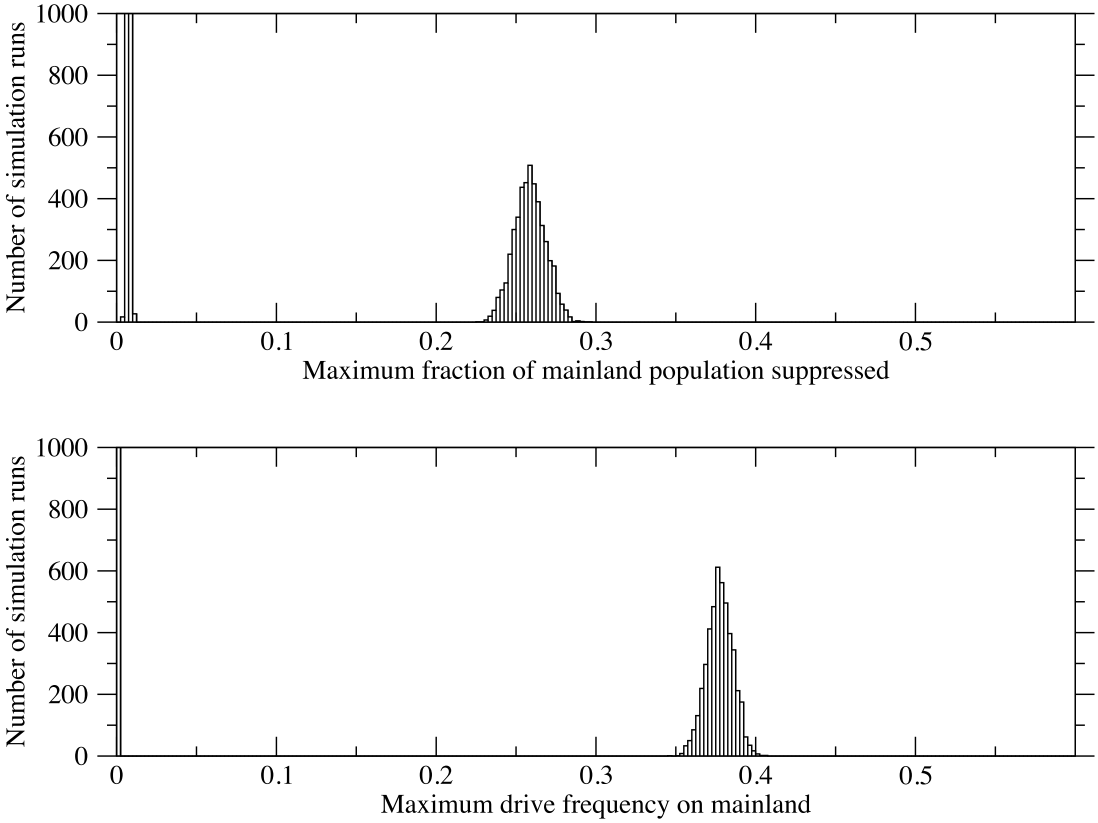

S.5. Population Suppression and Population Replacement

We conclude with a brief description of the dynamics of the model in the case where the island population is suppressed but not eliminated. We assume that drive bears a positive fitness cost, but that the cost imposed (either at fixation or at the polymorphic equilibrium, depending on the outcome of the population genetics) is not sufficient to lead to elimination. This setting can either describe a drive that is intended to suppress the island population or one that is designed as a population replacement strategy. (Expressions for the threshold conditions governing the population genetic outcome are given in[2,7], as are the allele frequencies at the polymorphic equilibrium (if it exists). Alphey and Bonsall[7] further provide threshold conditions for the elimination of a population given the population genetic outcome.)

The primary difference in the dynamics here is that drive will remain in the island population, and hence there will be continual introduction of drive to the mainland by migration. Given that drive is outcompeted on the mainland, this leads to the establishment of a polymorphic equilibrium between drive, resistant and wild-types, with the level of drive typically low. We note that if the drive fitness cost is low, these dynamics, specifically the reduction in the level of drive that occurs following its initial transient rise, can take a long time to play out.

Figure S.12 shows typical time series of the dynamics of the LFA model with a suppression drive that does not achieve elimination. Figure S.13 shows variation in the maximum level of suppression seen on the mainland, maximum mainland drive frequency, and long-term mainland drive frequency (at t=100 years) over a region of drive parameter space. (Note that we restrict the fitness cost s to be greater than 0.1 in order for our 100 year timescale to be appropriate to capture dynamics: as mentioned above, very low fitness costs lead to a very long timescale for the loss of drive on the mainland.) Figure S.14 explores the variation in the same quantities for the baseline suppression drive parameters, over a range of frequencies of resistance on the mainland and levels of migration. Results are also shown for a second set of drive parameters, with a lower fitness cost (s=0.3, h=0.3).

Supplemental Figure 12. Suppression/replacement dynamics. Top Panel: Island dynamics, showing relative population size (blue solid curve; left axis) and drive allele frequency (red dashed curve; right axis). Middle Panel: Mainland dynamics: relative population size (blue solid curve; left axis), and drive and resistant allele frequencies (red dashed and red dot-dashed curves, respectively; right axis). Bottom Panel: allele frequencies as in the previous panel, but depicted on a logarithmic scale. A drive that does not achieve elimination on the island is deployed (*s*=0.6 and *h*=0.3). For these parameters, the drive achieves fixation on the island but the resulting genetic load is not sufficient to cause elimination. This leads to a continual migration of drive-bearing individuals to the mainland. Initially, dynamics on the mainland play out much as seen for an elimination drive. However, the continual reintroduction of drive to the mainland by the migrants from the island leads to a polymorphic equilibrium for which drive is present at a low level (most visible on lower panel). Other parameters are as in Figure 3 of the main text, with homing probability of 0.95, initial resistance allele frequency of 95% on the mainland and per-capita migration rate of 0.012/year.

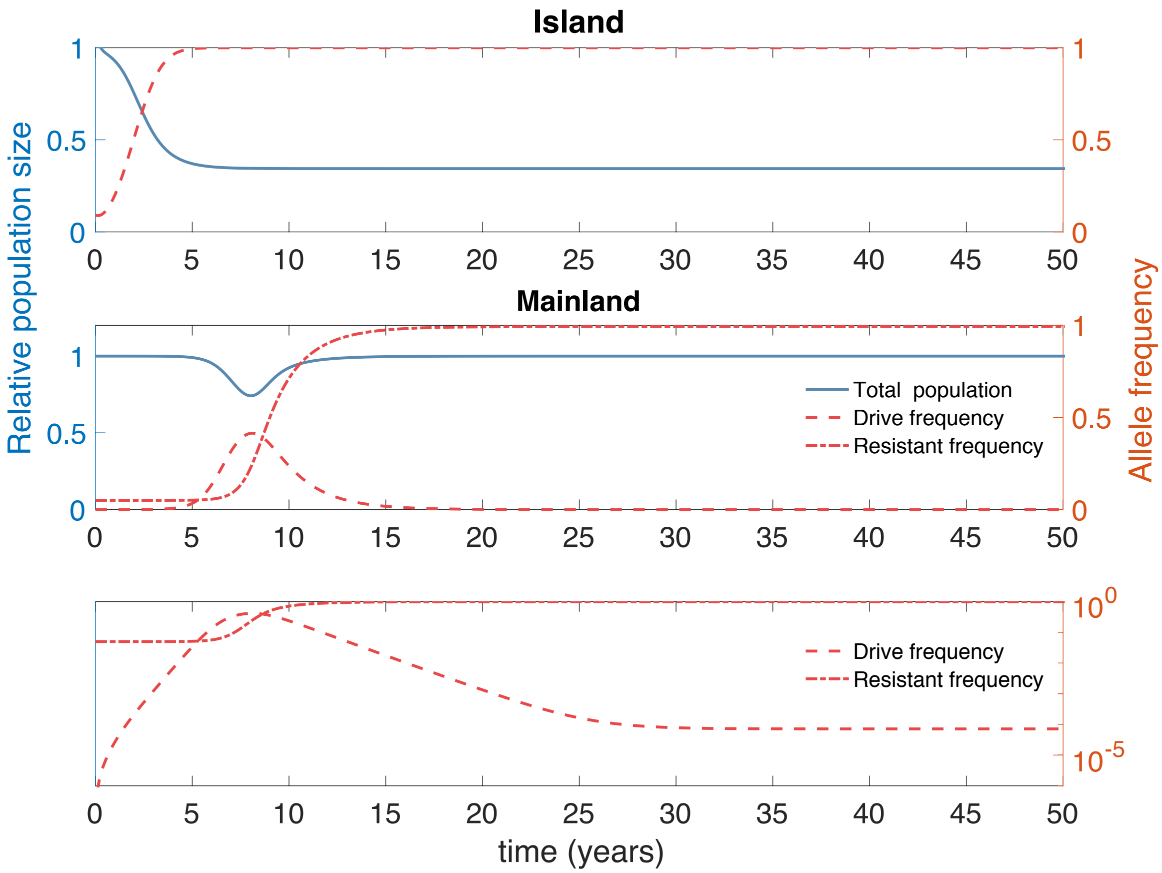

Supplemental Figure 13. Heatmaps showing the dependence of (panel a) the maximum suppression observed on the mainland, (panel b) the maximum level of drive seen on the mainland, and (panel c) the drive frequency on the mainland after 100 years, on drive fitness parameters *s* and *h* over regions of parameter space for which the drive does not have an invasion probability and suppresses the island population, but does not does not lead to extinction. The black asterisk denotes the drive parameters used in Figures S.12. All other parameters are as in Figure S.12. The scales on the color bars in panels (a) and (b) are chosen to be the same as in Figures 4 and 5 of the main text. White regions on this figure denote combinations of drive parameters that either lead to threshold behavior, loss of drive or for which the drive leads to elimination of the island population. The dashed curve denotes the boundary between the region of parameter space where drive approaches fixation (to the left of the curve) and where drive approaches a polymorphic equilibrium with wild-type (to the right of the curve). (Note that with the homing probability of 0.95 used here, the dashed curve lies in the region of drive space that leads to extinction of the island population.)

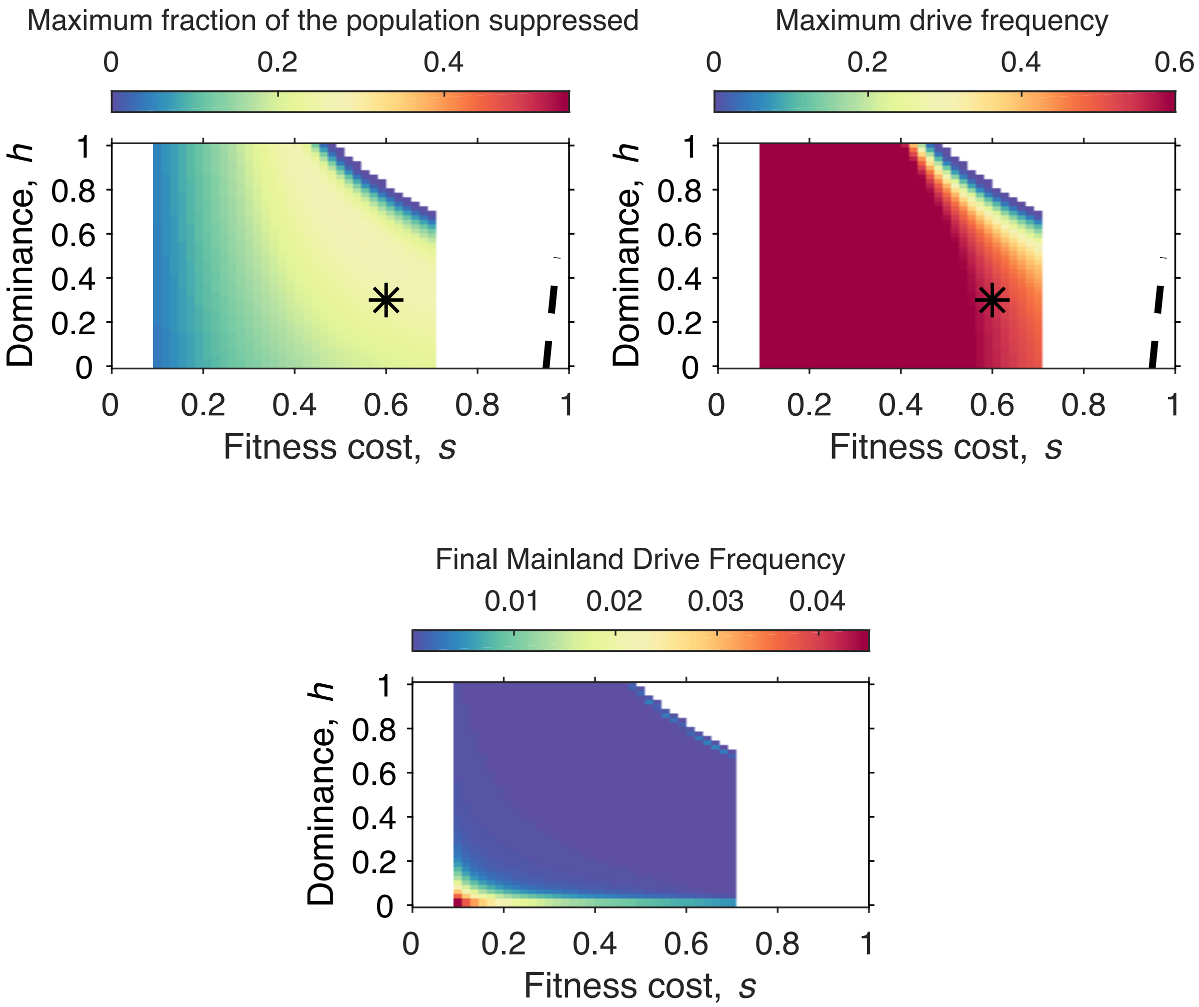

Supplemental Figure 14. Heatmaps showing the dependence of (panel a) the maximum suppression observed on the mainland, (panel b) the maximum level of drive seen on the mainland, and (panel c) the drive frequency on the mainland after 100 years, on initial level of resistance on the mainland and the migration rate, for drive parameters *s*=0.6 and *h*=0.3 that lead to threshold-free suppression but not extinction of the island population. Panels (d), (e), and (f) show the same information but for a drive with *s*=0.3 and h=0.3. The scales on the color bars in panels (a) and (d), and (b) and (e) are chosen to be the same as in Figures 4 and 5 of the main text. All other parameters are as in Figure S.12.

(a)

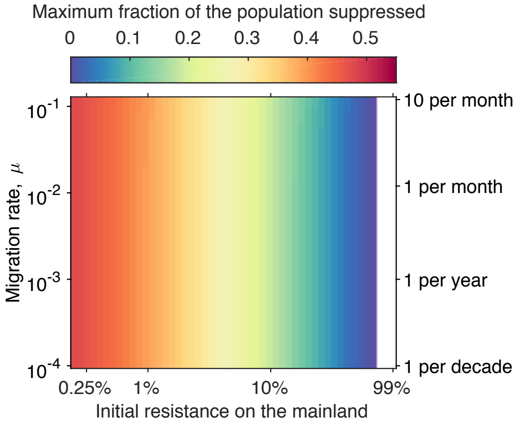

(b)

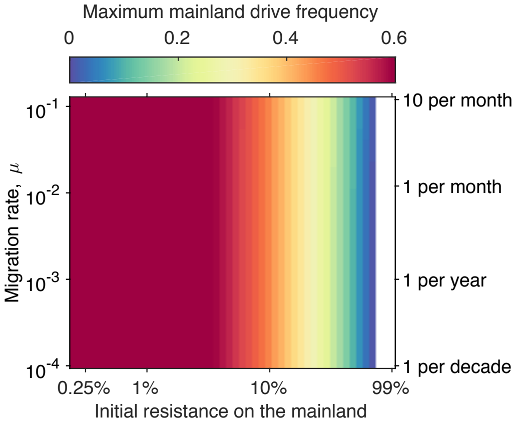

(c)

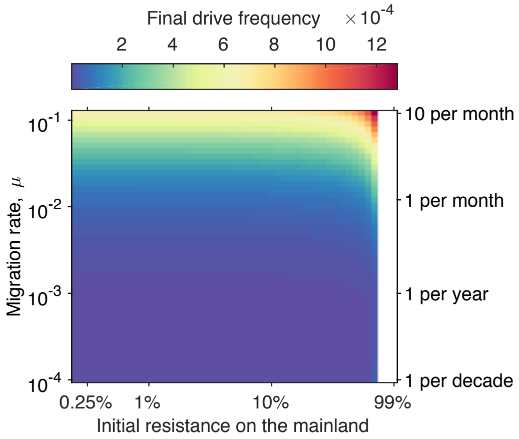

(Figure continues on next page)

(d)

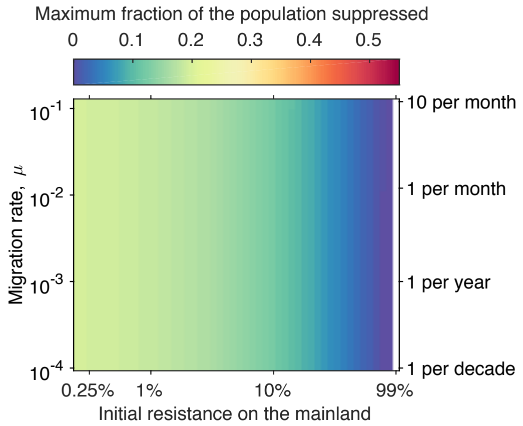

Maximum fraction of the population suppressed
0
0.1
0.2
0.3
0.4
0.5
10^{-1}
10^{-2}
10^{-3}
10^{-4}
Migration rate, μ
10 per month
1 per month
1 per year
1 per decade
0.25%
1%
10%
99%
Initial resistance on the mainland

(e)

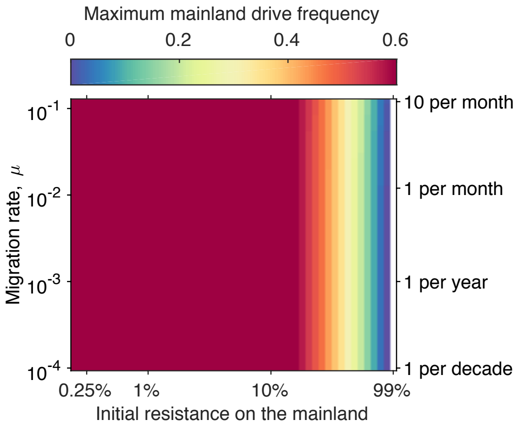

Maximum mainland drive frequency
0
0.2
0.4
0.6
10^{-1}
10^{-2}
10^{-3}
10^{-4}
Migration rate, μ
10 per month
1 per month
1 per year
1 per decade
0.25%
1%
10%
99%
Initial resistance on the mainland

(f)

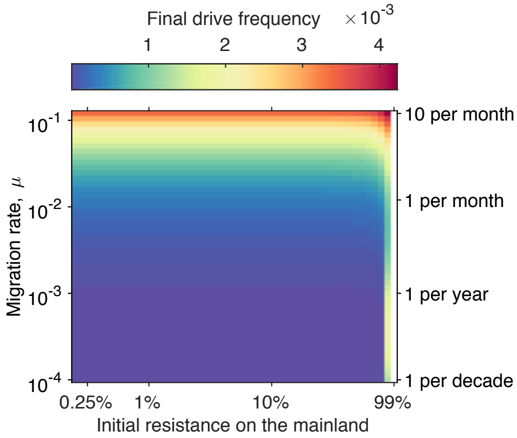

Final drive frequency
$\times 10^{-3}$
1
2
3
4
10^{-1}
10^{-2}
10^{-3}
10^{-4}
Migration rate, μ
10 per month
1 per month
1 per year
1 per decade
0.25%
1%
10%
99%
Initial resistance on the mainland

References:

1. Strogatz, S. H. *Nonlinear Dynamics and Chaos.* (Westview Press, 2014).

2. Deredec, A., Burt, A. & Godfray, C. The population genetics of using homing endonuclease genes (HEGs) in vector and pest management. *Genetics* **179**, 2013-2026 (2008).

3. Vella, M. R., Gunning, C. E., Lloyd, A. L. & Gould, F. Evaluating strategies for reversing CRISPR-Cas9 gene drives. *Scientific Reports* **7**, 11038 (2017).

4. Prowse, T. A. A., Adikusuma, F., Cassey, P., Thomas, P. & Ross, J. V. A Y-chromosome shredding gene drive for controlling pest vertebrate populations. *eLife* **8**, e41873 (2019).

5. Saltelli, A. *et al. Global Sensitivity Analysis, the Primer*. (Wiley, 2008).

6. Pianosi, F., Sarrazin, F. & Wagener, T. A Matlab toolbox for global sensitivity analysis. *Environ. Model. Software* **70**, 80-85 (2015).

7. Alphey, N. & Bonsall, M. B. Interplay of population genetics and dynamics in the genetic control of mosquitoes. *J. R. Soc. Interface* **11**, 20131071 (2014).

8. DeAngelis, D. L. & Waterhouse, J. C. Equilibrium and Nonequilibrium Concepts in Ecological Models. *Ecol. Monogr.* **57**, 1-21 (1987).

9. Benton, T. G. & Grant, A. Elasticity analysis as an important tool in evolutionary and population ecology. *Trends Ecol. Evol.* **14**, 467-471 (1999).

10. Robert, M. A., Okamoto, K., Lloyd, A. L. & Gould, F. A reduce and replace strategy for suppressing vector-borne diseases: insights from a deterministic model. *PLoS One* **8**, e73233 (2013).

11. Prowse, T. A. A. *et al.* Dodging silver bullets: good CRISPR gene-drive design is critical for eradicating exotic vertebrates. *Proc. R. Soc. Lond. B* **284**, 20170799 (2017).

12. Backus, G. A., & Gross, K. Genetic engineering to eradicate invasive mice on islands: modeling the efficiency and ecological impacts. *Ecosphere* **7**, e01589 (2016).

13. Renshaw, E. *Modelling Biological Populations in Space and Time*. (Cambridge University Press, 1991).

14. Marshall, J. M. The effect of gene drive on containment of transgenic mosquitoes. *J. Theor. Biol.* **258**, 250-265 (2009).
