# Supplementary material for: Locally Fixed Alleles: A method to localize gene drive to island populations
Source: Sci Rep. 2019 Nov 1;9:15821. doi: 10.1038/s41598-019-51994-0 (PMC6825234; doi:10.1038/s41598-019-51994-0)
Supplement: Supplementary file 3 — Dataset 2 [file 41598_2019_51994_MOESM3_ESM.pdf]

Supplementary Information: Maple Worksheet printout for

Locally Fixed Alleles: A method to localize gene drive to island populations

Jaye Sudweeks [a], Brandon Hollingsworth [b], Dimitri V. Blondel [c], Karl J. Campbell [d], Sumit Dhole [e], John D. Eisemann [f], Owain Edwards [g], John Godwin [c,h], Gregg R. Howald [d], Kevin P. Oh [f,i], Antoinette J. Piaggio [f], Thomas A. A. Prowse [j], Joshua V. Ross [j], J. Royden Saah [d,h], Aaron B. Shiels [f], Paul Q. Thomas [k], David W. Threadgill [l], Michael R. Vella [b], Fred Gould [e,h] and Alun L. Lloyd [a,b,*]

(a) Department of Mathematics, North Carolina State University, Raleigh, NC 27695, USA
(b) Biomathematics Graduate Program, North Carolina State University, Raleigh, NC 27695, USA
(c) Department of Biological Sciences, North Carolina State University, Raleigh, NC 27695, USA
(d) Island Conservation, 2100 Delaware Ave., Suite 1, Santa Cruz, CA 95060 USA
(e) Department of Entomology and Plant Pathology, North Carolina State University, Raleigh, NC 27695, USA
(f) National Wildlife Research Center, US Department of Agriculture, Fort Collins, CO 80521, USA
(g) CSIRO Land & Water, Centre for Environment and Life Sciences, Floreat, WA, Australia
(h) Genetic Engineering and Society Center, North Carolina State University, Raleigh, NC 27695, USA
(i) Department of Microbiology, Immunology & Pathology, Colorado State University, Fort Collins, CO 80523, USA
(j) School of Mathematical Sciences, The University of Adelaide, Adelaide, SA 5005, Australia
(k) The Robinson Research Institute and School of Medicine, The University of Adelaide, Adelaide, SA 5005, Australia
(l) Department of Molecular and Cellular Medicine, Texas A&M University, College Station, TX 77843, USA

* Corresponding author, email: alun_lloyd@ncsu.edu

```
> # P(i,j,k) calculations for homing with probability e, sensitive-wildtype (S), drive (D) and resistant
     (R)
> # genotypes:
> # 1 : RR , 2: SS, 3: DD, 4: SR, 5: DR, 6: SD
>
> P := Array(1 ..6, 1 ..6, 1 ..6, fill = infinity);
```

$$P := \begin{bmatrix} \text{1..6 x 1..6 x 1..6 Array} \\ \text{Data Type: anything} \\ \text{Storage: rectangular} \\ \text{Order: Fortran\_order} \end{bmatrix} \qquad (1)$$

```
> # RRxRR ( j=1, k=1)
> # all offspring are RR
> P(1, 1, 1) := 1 : P(2, 1, 1) := 0 : P(3, 1, 1) := 0 : P(4, 1, 1) := 0 : P(5, 1, 1) := 0 : P(6, 1,
     1) := 0 :
>
> #RRxSS (j=1,k=2)
> # all offspring are SR
> P(1, 1, 2) := 0 : P(2, 1, 2) := 0 : P(3, 1, 2) := 0 : P(4, 1, 2) := 1 : P(5, 1, 2) := 0 : P(6, 1,
     2) := 0 :
>
> #RRxDD (j=1,k=3)
> # all offspring are DR
> P(1, 1, 3) := 0 : P(2, 1, 3) := 0 : P(3, 1, 3) := 0 : P(4, 1, 3) := 0 : P(5, 1, 3) := 1 : P(6, 1,
     3) := 0 :
>
> #RRxSR (j=1,k=4)
> #half of offspring are RR, half are SR
> P(1, 1, 4) := 1/2 : P(2, 1, 4) := 0 : P(3, 1, 4) := 0 : P(4, 1, 4) := 1/2 : P(5, 1, 4) := 0 : P(6,
     1, 4) := 0 :
>
> #RRxDR (j=1,k=5)
> # no drive here because K is resistant
> # half of offspring are RR, half are DR
> P(1, 1, 5) := 1/2 : P(2, 1, 5) := 0 : P(3, 1, 5) := 0 : P(4, 1, 5) := 0 : P(5, 1, 5) := 1/2 : P(6,
     1, 5) := 0 :
>
> #RRxSD (j=1,k=6)
> # with probability e, SD becomes DD in terms of alleles produced; with prob (1-e) remains SD
> # prob e: RRxDD: all offspring are DR
> # prob (1-e): RRxSD: half of offspring are DR, half are SR
```

> $P(1,1,6) := 0 : P(2,1,6) := 0 : P(3,1,6) := 0 : P(4,1,6) := (1-e)\cdot\left(\frac{1}{2}\right) : P(5,1,6) := e + (1-e)\cdot\left(\frac{1}{2}\right) : P(6,1,6) := 0 :$

>

> *#SSxRR (j=2,k=1)*

> *#all offspring are SR*

> $P(1,2,1) := 0 : P(2,2,1) := 0 : P(3,2,1) := 0 : P(4,2,1) := 1 : P(5,2,1) := 0 : P(6,2,1) := 0 :$

>

> *#SSxSS (j=2,k=2)*

> *#all offspring are SS*

> $P(1,2,2) := 0 : P(2,2,2) := 1 : P(3,2,2) := 0 : P(4,2,2) := 0 : P(5,2,2) := 0 : P(6,2,2) := 0 :$

>

> *#SSxDD (j=2,k=3)*

> *# all offspring are SD*

> $P(1,2,3) := 0 : P(2,2,3) := 0 : P(3,2,3) := 0 : P(4,2,3) := 0 : P(5,2,3) := 0 : P(6,2,3) := 1 :$

>

> *#SSxSR (j=2,k=4)*

> *#half of offspring are SR, half are SS*

> $P(1,2,4) := 0 : P(2,2,4) := \frac{1}{2} : P(3,2,4) := 0 : P(4,2,4) := \frac{1}{2} : P(5,2,4) := 0 : P(6,2,4) := 0 :$

>

> *#SSxDR (j=2,k=5)*

> *#no drive here, so half of offspring are SR, half are SD*

> $P(1,2,5) := 0 : P(2,2,5) := 0 : P(3,2,5) := 0 : P(4,2,5) := \frac{1}{2} : P(5,2,5) := 0 : P(6,2,5) := \frac{1}{2} :$

>

> *#SSxSD (j=2,k=6)*

> *# prob e this is SSxDD, all of whose offspring will be SD*

> *# prob (1-e) this is SSxSD, half of whose offspring will be SS, half of offspring SD*

> $P(1,2,6) := 0 : P(2,2,6) := (1-e)\cdot\left(\frac{1}{2}\right) : P(3,2,6) := 0 : P(4,2,6) := 0 : P(5,2,6) := 0 : P(6,2,6) := e + (1-e)\cdot\left(\frac{1}{2}\right) :$

>

> *#DDxRR (j=3,k=1)*

> *#all offspring are DR*

> $P(1,3,1) := 0 : P(2,3,1) := 0 : P(3,3,1) := 0 : P(4,3,1) := 0 : P(5,3,1) := 1 : P(6,3,1) := 0 :$

>

> *#DDxSS (j=3,k=2)*

> *#all offspring are SD*

> $P(1,3,2) := 0 : P(2,3,2) := 0 : P(3,3,2) := 0 : P(4,3,2) := 0 : P(5,3,2) := 0 : P(6,3,2) := 1 :$

>

> *#DDxDD (j=3,k=3)*

> *#all offspring are DD*

> $P(1,3,3) := 0 : P(2,3,3) := 0 : P(3,3,3) := 1 : P(4,3,3) := 0 : P(5,3,3) := 0 : P(6,3,3) := 0 :$

>

> *#DDxSR (j=3,k=4)*

> *#half of offspring are DR, half are SD*

> $P(1,3,4) := 0 : P(2,3,4) := 0 : P(3,3,4) := 0 : P(4,3,4) := 0 : P(5,3,4) := \frac{1}{2} : P(6,3,4) := \frac{1}{2} :$

>

> *#DDxDR (j=3,k=5)*

> *#no drive here, so half of offspring are DR, half are DD*

> $P(1,3,5) := 0 : P(2,3,5) := 0 : P(3,3,5) := \frac{1}{2} : P(4,3,5) := 0 : P(5,3,5) := \frac{1}{2} : P(6,3,5) := 0 :$

>

> *#DDxSD (j=3,k=6)*

> *#with prob e this is DDxDD, for which all offspring are DD*

> *#with prob (1-e) this is DDxSD, for which half of offspring are DD, half are SD*

> $P(1,3,6) := 0 : P(2,3,6) := 0 : P(3,3,6) := e + (1-e)\cdot\left(\frac{1}{2}\right) : P(4,3,6) := 0 : P(5,3,6) := 0 : P(6,3,6) := (1-e)\cdot\left(\frac{1}{2}\right) :$

>

> *#halfway through!*

>

> *#SRxRR (j=4,k=1)*

> *# half of offspring are SR, half RR*

> $P(1,4,1) := \frac{1}{2} : P(2,4,1) := 0 : P(3,4,1) := 0 : P(4,4,1) := \frac{1}{2} : P(5,4,1) := 0 : P(6,4,1) := 0 :$

>

> *#SRxSS (j=4,k=2)*

> #half of offspring are SR, half SS

> $P(1,4,2) := 0 : P(2,4,2) := \frac{1}{2} : P(3,4,2) := 0 : P(4,4,2) := \frac{1}{2} : P(5,4,2) := 0 : P(6, 4,2) := 0 :$

>

> #SRxDD (j=4,k=3)

> #half of offspring are DR, half are SD

> $P(1,4,3) := 0 : P(2,4,3) := 0 : P(3,4,3) := 0 : P(4,4,3) := 0 : P(5,4,3) := \frac{1}{2} : P(6, 4,3) := \frac{1}{2} :$

>

> #SRxSR (j=4, k=4)

> #quarter RR, half SR, quarter SS

> $P(1,4,4) := \frac{1}{4} : P(2,4,4) := \frac{1}{4} : P(3,4,4) := 0 : P(4,4,4) := \frac{1}{2} : P(5,4,4) := 0 : P(6,4,4) := 0 :$

>

> #SRxDR (j=4,k=5)

> # no drive here

> #quarter are RR, quarter are DR, quarter are SR, quarter are SD

> $P(1,4,5) := \frac{1}{4} : P(2,4,5) := 0 : P(3,4,5) := 0 : P(4,4,5) := \frac{1}{4} : P(5,4,5) := \frac{1}{4} : P(6,4,5) := \frac{1}{4} :$

>

> #SRxSD (j=4,k=6)

> # with prob e this is SRxDD, for which half are DR, half SD

> # with prob (1-e) this is SRxSD, for which quarter are DR, quarter are SR, quarter are SD, quarter are SS

> $P(1,4,6) := 0 : P(2,4,6) := (1-e)\cdot\left(\frac{1}{4}\right) : P(3,4,6) := 0 : P(4,4,6) := (1-e)\cdot\left(\frac{1}{4}\right) : P(5,4,6) := e\cdot\left(\frac{1}{2}\right) + (1-e)\cdot\left(\frac{1}{4}\right) : P(6,4,6) := e\cdot\left(\frac{1}{2}\right) + (1-e)\cdot\left(\frac{1}{4}\right) :$

>

> #DRxRR (j=5,k=1)

> #no drive here

> # half are RR, half are DR

> $P(1,5,1) := \frac{1}{2} : P(2,5,1) := 0 : P(3,5,1) := 0 : P(4,5,1) := 0 : P(5,5,1) := \frac{1}{2} : P(6, 5,1) := 0 :$

>

> *#DRxSS (j=5,k=2)*

> *#half are SR, half are SD*

> $P(1,5,2) := 0 : P(2,5,2) := 0 : P(3,5,2) := 0 : P(4,5,2) := \frac{1}{2} : P(5,5,2) := 0 : P(6,5,2) := \frac{1}{2} :$

>

> *#DRxDD (j=5,k=3)*

> *#half are DR, half are DD*

> $P(1,5,3) := 0 : P(2,5,3) := 0 : P(3,5,3) := \frac{1}{2} : P(4,5,3) := 0 : P(5,5,3) := \frac{1}{2} : P(6,5,3) := 0 :$

>

> *#DRxSR (j=5,k=4)*

> *#quarter are RR, quarter DR, quarter SD, quarter SR*

> $P(1,5,4) := \frac{1}{4} : P(2,5,4) := 0 : P(3,5,4) := 0 : P(4,5,4) := \frac{1}{4} : P(5,5,4) := \frac{1}{4} : P(6,5,4) := \frac{1}{4} :$

>

> *#DRxDR (j=5,k=5)*

> *#quarter are RR, quarter are DD, half are DR*

> $P(1,5,5) := \frac{1}{4} : P(2,5,5) := 0 : P(3,5,5) := \frac{1}{4} : P(4,5,5) := 0 : P(5,5,5) := \frac{1}{2} : P(6,5,5) := 0 :$

>

> *#DRxSD (j=5,k=6)*

> *#can have drive here*

> *# with prob e we have DRxDD, for which half are DR, half are DD*

> *#with prob (1-e) we have DRxSD, for which quarter are DR, quarter DD, quarter SD, quarter SR*

> $P(1,5,6) := 0 : P(2,5,6) := 0 : P(3,5,6) := e\cdot\left(\frac{1}{2}\right) + (1-e)\cdot\left(\frac{1}{4}\right) : P(4,5,6) := (1-e)\cdot\left(\frac{1}{4}\right) : P(5,5,6) := e\cdot\left(\frac{1}{2}\right) + (1-e)\cdot\left(\frac{1}{4}\right) : P(6,5,6) := (1-e)\cdot\left(\frac{1}{4}\right) :$

>

> *#SDxRR (j=6,k=1)*

> *#homing here*

> *#with prob e we have DDxRR, for which all offspring are DR*

> *#with prob (1-e) we have SDxRR, for which half are DR, half SR*

> $P(1,6,1) := 0 : P(2,6,1) := 0 : P(3,6,1) := 0 : P(4,6,1) := (1-e)\cdot\left(\frac{1}{2}\right) : P(5,6,$

$1) := e + (1-e)\cdot\left(\frac{1}{2}\right) : P(6, 6, 1) := 0 :$

>

> *#SDxSS (j=6,k=2)*

> *#with prob e we have DDxSS, for which all offspring are SD*

> *#with prob 1-e we have SDxSS, for which half are SD, half SS*

> $P(1, 6, 2) := 0 : P(2, 6, 2) := (1-e)\cdot\left(\frac{1}{2}\right) : P(3, 6, 2) := 0 : P(4, 6, 2) := 0 : P(5, 6, 2) := 0 : P(6, 6, 2) := e + (1-e)\cdot\left(\frac{1}{2}\right) :$

>

> *#SDxDD (j=6,k=3)*

> *#with prob e we have DDxDD, for which all are DD*

> *#prob (1-e) we have SDxDD, for which half are SD, half are DD*

> $P(1, 6, 3) := 0 : P(2, 6, 3) := 0 : P(3, 6, 3) := e + (1-e)\cdot\left(\frac{1}{2}\right) : P(4, 6, 3) := 0 : P(5, 6, 3) := 0 : P(6, 6, 3) := (1-e)\cdot\left(\frac{1}{2}\right) :$

>

> *#SDxSR (j=6,k=4)*

> *#with prob e we have DDxSR, for which half are DR, half SD*

> *#with prob (1-e) we have SDxSR, for which quarter are DR, quarter are SD, quarter SR, quarter SS*

> $P(1, 6, 4) := 0 : P(2, 6, 4) := (1-e)\cdot\left(\frac{1}{4}\right) : P(3, 6, 4) := 0 : P(4, 6, 4) := (1-e)\cdot\left(\frac{1}{4}\right) : P(5, 6, 4) := e\cdot\left(\frac{1}{2}\right) + (1-e)\cdot\left(\frac{1}{4}\right) : P(6, 6, 4) := e\cdot\left(\frac{1}{2}\right) + (1-e)\cdot\left(\frac{1}{4}\right) :$

>

> *#SDxDR (j=6,k=5)*

> *#prob e we have DDxDR, for which half are DR, half DD*

> *#prob (1-e) SDxDR, for which quarter are DR, quarter DD, quarter SR, quarter SD*

> $P(1, 6, 5) := 0 : P(2, 6, 5) := 0 : P(3, 6, 5) := e\cdot\left(\frac{1}{2}\right) + (1-e)\cdot\left(\frac{1}{4}\right) : P(4, 6, 5) := (1-e)\cdot\left(\frac{1}{4}\right) : P(5, 6, 5) := e\cdot\left(\frac{1}{2}\right) + (1-e)\cdot\left(\frac{1}{4}\right) : P(6, 6, 5) := (1-e)\cdot\left(\frac{1}{4}\right) :$

>

> *#SDxSD (j=6,k=6)*

> *#with prob* $(e)^2$ *, homing occurs at both* **and** *so we have DDxDD : all offspring DD*

> *#with prob* $2\cdot e\cdot(1\text{-}e)$*, we have homing at exactly one, so we have SDxDD: half are DD, half are SD*

> *#with prob* $(1\text{-}e)^2$ *we have homing at neither, so SDxSD, meaning quarter are DD,*

quarter are SS, half are SD

```
> P(1, 6, 6) := 0 : P(2, 6, 6) := (1 − e)^2·(1/4) : P(3, 6, 6) := (e)^2 + 2·e·(1 − e)·(1/2)
    + (1 − e)^2·(1/4) : P(4, 6, 6) := 0 : P(5, 6, 6) := 0 : P(6, 6, 6) := 2·e·(1 − e)·(1/2)
    + (1 − e)^2·(1/2) :
>
> w := Array(1 ..6) :
> w(1) := 1 : w(2) := 1 : w(3) := 1 − s : w(4) := 1 : w(5) := 1 − h·s : w(6) := 1 − h·s;
```

$$w := \left[\begin{array}{cccccc} 1 & 1 & 1-s & 1 & -h\,s+1 & -h\,s+1 \end{array}\right] \quad \textbf{(2)}$$

```
>
> N_tot := N[1] + N[2] + N[3] + N[4] + N[5] + N[6] :
  for i from 1 to 6 do
  b[i] := 0 :
  for j from 1 to 6 do
  for k from 1 to 6 do
  b[i] := b[i] + P(i, j, k)·N[j]·N[k] :
  end do:
  end do:
  b[i] := b[i]·lambda·w(i) / N_tot;
  end do:
>
> f := i→b[i]·(1 − q·N_tot) − rho·N[i] − alpha·N[i]·N_tot :
>
> odes := [f(1), f(2), f(3), f(4), f(5), f(6)];
```

$$odes := \left[\frac{1}{N_1+N_2+N_3+N_4+N_5+N_6}\left(\left(N_1^2+N_1\,N_4+N_1\,N_5+\frac{1}{4}\,N_4^2+\frac{1}{2}\,N_4\,N_5+\frac{1}{4}\,N_5^2\right)\lambda\left(-\left(N_1+N_2+N_3+N_4+N_5+N_6\right)q+1\right)\right)-\rho\,N_1-\alpha\,N_1\left(N_1+N_2+N_3+N_4+N_5+N_6\right), \frac{1}{N_1+N_2+N_3+N_4+N_5+N_6}\left(\left(N_2^2+N_2\,N_4+2\left(\frac{1}{2}-\frac{e}{2}\right)N_2\,N_6+\frac{N_4^2}{4}+2\left(\frac{1}{4}-\frac{e}{4}\right)N_4\,N_6+\frac{(1-e)^2\,N_6^2}{4}\right)\lambda\left(-\left(N_1+N_2+N_3\right.\right.\right.\right. \quad \textbf{(3)}$$

$$+ N_4 + N_5 + N_6) q + 1) \Big) - \rho N_2 - \alpha N_2 (N_1 + N_2 + N_3 + N_4 + N_5 + N_6),$$

$$\frac{1}{N_1 + N_2 + N_3 + N_4 + N_5 + N_6} \Bigg(\Bigg(N_3^2 + N_3 N_5 + 2 \left(\frac{e}{2} + \frac{1}{2} \right) N_3 N_6 + \frac{N_5^2}{4} + 2 \left(\frac{e}{4} \right.$$

$$\left. + \frac{1}{4} \right) N_5 N_6 + \left(e^2 + e (1 - e) + \frac{(1 - e)^2}{4} \right) N_6^2 \Bigg) \lambda (1 - s) (- (N_1 + N_2 + N_3 + N_4$$

$$+ N_5 + N_6) q + 1) \Bigg) - \rho N_3 - \alpha N_3 (N_1 + N_2 + N_3 + N_4 + N_5 + N_6),$$

$$\frac{1}{N_1 + N_2 + N_3 + N_4 + N_5 + N_6} \Bigg(\Bigg(2 N_1 N_2 + N_1 N_4 + 2 \left(\frac{1}{2} - \frac{e}{2} \right) N_1 N_6 + N_2 N_4$$

$$+ N_2 N_5 + \frac{N_4^2}{2} + \frac{N_4 N_5}{2} + 2 \left(\frac{1}{4} - \frac{e}{4} \right) N_4 N_6 + 2 \left(\frac{1}{4} - \frac{e}{4} \right) N_5 N_6 \Bigg) \lambda (- (N_1$$

$$+ N_2 + N_3 + N_4 + N_5 + N_6) q + 1) \Bigg) - \rho N_4 - \alpha N_4 (N_1 + N_2 + N_3 + N_4 + N_5$$

$$+ N_6), \frac{1}{N_1 + N_2 + N_3 + N_4 + N_5 + N_6} \Bigg(\Bigg(2 N_1 N_3 + N_1 N_5 + 2 \left(\frac{e}{2} + \frac{1}{2} \right) N_1 N_6$$

$$+ N_3 N_4 + N_3 N_5 + \frac{N_4 N_5}{2} + 2 \left(\frac{e}{4} + \frac{1}{4} \right) N_4 N_6 + \frac{N_5^2}{2} + 2 \left(\frac{e}{4} + \frac{1}{4} \right) N_5 N_6 \Bigg) \lambda ($$

$$- h s + 1) (- (N_1 + N_2 + N_3 + N_4 + N_5 + N_6) q + 1) \Bigg) - \rho N_5 - \alpha N_5 (N_1 + N_2 + N_3$$

$$+ N_4 + N_5 + N_6), \frac{1}{N_1 + N_2 + N_3 + N_4 + N_5 + N_6} \Bigg(\Bigg(2 N_2 N_3 + N_2 N_5 + 2 \left(\frac{e}{2} \right.$$

$$\left. + \frac{1}{2} \right) N_2 N_6 + N_3 N_4 + 2 \left(\frac{1}{2} - \frac{e}{2} \right) N_3 N_6 + \frac{N_4 N_5}{2} + 2 \left(\frac{e}{4} + \frac{1}{4} \right) N_4 N_6$$

$$+ 2\left(\frac{1}{4} - \frac{e}{4}\right) N_5 N_6 + \left(e(1-e) + \frac{(1-e)^2}{2}\right) N_6^2\Bigg) \lambda(-h s + 1)\Big(-\big(N_1 + N_2 + N_3 + N_4 + N_5 + N_6\big) q + 1\Big)\Bigg) - \rho N_6 - \alpha N_6 \left(N_1 + N_2 + N_3 + N_4 + N_5 + N_6\right)\Bigg]$$

\>

\> $equil\_N := \frac{\lambda - \rho}{\lambda q + \alpha}$;

$$equil\_N := \frac{\lambda - \rho}{\lambda q + \alpha} \tag{4}$$

\>

\> with(VectorCalculus) : with(LinearAlgebra) :

\>

\> J := Jacobian([odes[1], odes[2], odes[3], odes[4], odes[5], odes[6]], [N[1], N[2], N[3], N[4], N[5], N[6]]) :

\> # Evaluate Jacobian matrix at Wildtype:Resistant equilibrium, with resistant allele frequency equal to q_R

Keep in mind that there is a line of Wildtype:Resistant equilibria, as we have no cost of resistance here

\> J_WT_RES := simplify(subs(N[3] = 0, N[5] = 0, N[6] = 0, N[1] = equil_N·q_R·q_R, N[2] = equil_N·(1 − q_R)·(1 − q_R), N[4] = equil_N·2·q_R·(1 − q_R), J));

$$J\_WT\_RES := \Bigg[\tag{5}$$

$$\Bigg[\frac{-q\_R^2 \lambda^2 q + \left(\left(-2 q\_R^2 + 2 q\_R - 1\right)\alpha + 2 q \left(q\_R - \frac{1}{2}\right)\rho\right)\lambda + \alpha q\_R^2 \rho}{\lambda q + \alpha},$$

$$-\frac{q\_R^2 \left((2\lambda - \rho)\alpha + \lambda^2 q\right)}{\lambda q + \alpha}, -\frac{q\_R^2 \left((2\lambda - \rho)\alpha + \lambda^2 q\right)}{\lambda q + \alpha},$$

$$-\frac{q\_R\left(\lambda^2 q\, q\_R + \left((2 q\_R - 1)\alpha - q\rho\right)\lambda - \alpha q\_R \rho\right)}{\lambda q + \alpha},$$

$$-\frac{q\_R\left(\lambda^2 q\, q\_R + \left((2 q\_R - 1)\alpha - q\rho\right)\lambda - \alpha q\_R \rho\right)}{\lambda q + \alpha},$$

$$-\frac{q\_R^2 \left((2\lambda - \rho)\alpha + \lambda^2 q\right)}{\lambda q + \alpha}\Bigg],$$

$$\Bigg[-\frac{\left((2\lambda - \rho)\alpha + \lambda^2 q\right)(-1 + q\_R)^2}{\lambda q + \alpha}, \frac{1}{\lambda q + \alpha}\Big(-(-1 + q\_R)^2 q \lambda^2 + \Big(\Big($$

$$-2\,q\_R^2 + 2\,q\_R - 1)\,\alpha - 2\,q\left(q\_R - \frac{1}{2}\right)\rho\Big)\,\lambda + \rho\,\alpha\,(-1 + q\_R)^2\Big),$$

$$-\frac{\left((2\,\lambda - \rho)\,\alpha + \lambda^2\,q\right)\,(-1 + q\_R)^2}{\lambda\,q + \alpha},$$

$$-\frac{\left(q\,(-1 + q\_R)\,\lambda^2 + \left((2\,q\_R - 1)\,\alpha + q\,\rho\right)\lambda - \alpha\,\rho\,(-1 + q\_R)\right)\,(-1 + q\_R)}{\lambda\,q + \alpha},$$

$$-\frac{\left((2\,\lambda - \rho)\,\alpha + \lambda^2\,q\right)\,(-1 + q\_R)^2}{\lambda\,q + \alpha},\ \frac{1}{\lambda\,q + \alpha}\Big((-1 + q\_R)\,\Big(-q\,(-1$$

$$+ q\_R)\,\lambda^2 + \left((e - 2\,q\_R + 1)\,\alpha + q\,\rho\,(-1 + e)\right)\lambda + \alpha\,\rho\,(-1 + q\_R)\Big)\Big)\Bigg],$$

$$\left[0,\ 0,\ -\frac{\lambda\,(q\,\rho + \alpha)}{\lambda\,q + \alpha},\ 0,\ 0,\ 0\right],$$

$$\Bigg[\frac{2\,(-1 + q\_R)\,\left(\lambda^2\,q\,q\_R + \left((2\,q\_R - 1)\,\alpha - q\,\rho\right)\lambda - \alpha\,q\_R\,\rho\right)}{\lambda\,q + \alpha},$$

$$\frac{2\,q\_R\left(q\,(-1 + q\_R)\,\lambda^2 + \left((2\,q\_R - 1)\,\alpha + q\,\rho\right)\lambda - \alpha\,\rho\,(-1 + q\_R)\right)}{\lambda\,q + \alpha},$$

$$\frac{2\,q\_R\left((2\,\lambda - \rho)\,\alpha + \lambda^2\,q\right)\,(-1 + q\_R)}{\lambda\,q + \alpha},$$

$$\frac{2\,q\_R\left((2\,\lambda - \rho)\,\alpha + \lambda^2\,q\right)\,(-1 + q\_R)}{\lambda\,q + \alpha},$$

$$\frac{(-1 + q\_R)\,\left(2\,\lambda^2\,q\,q\_R + 4\,\alpha\,\lambda\,q\_R - 2\,\alpha\,q\_R\,\rho - \lambda\,q\,\rho - \alpha\,\lambda\right)}{\lambda\,q + \alpha},$$

$$-\frac{1}{\lambda\,q + \alpha}\Big(q\_R\,\Big(-2\,q\,(-1 + q\_R)\,\lambda^2 + \left((e - 4\,q\_R + 3)\,\alpha + q\,\rho\,(-1 + e)\right)\lambda$$

$$+ 2\,\alpha\,\rho\,(-1 + q\_R)\Big)\Big)\Bigg],$$

$$\Bigg[0,\ 0,\ -\frac{2\,q\_R\,\lambda\,(h\,s - 1)\,(q\,\rho + \alpha)}{\lambda\,q + \alpha},\ 0,\ -\frac{\lambda\,(q\,\rho + \alpha)\,(h\,s\,q\_R - q\_R + 1)}{\lambda\,q + \alpha},$$

$$-\frac{q\_R\,(e + 1)\,\lambda\,(h\,s - 1)\,(q\,\rho + \alpha)}{\lambda\,q + \alpha}\Bigg],$$

$$\left[0, 0, \frac{2\,(-1+q\_R)\,\lambda\,(h\,s-1)\,(q\,\rho+\alpha)}{\lambda\,q+\alpha}, 0, \frac{(-1+q\_R)\,\lambda\,(h\,s-1)\,(q\,\rho+\alpha)}{\lambda\,q+\alpha}, \frac{\lambda\,((h\,s-1)\,(e+1)\,q\_R-e\,h\,s-h\,s+e)\,(q\,\rho+\alpha)}{\lambda\,q+\alpha}\right]\right]$$

> *simplify(Eigenvalues(J_WT_RES));*

$$\left[\begin{array}{c} 0 \\ -\lambda+\rho \\ \frac{((-1+q\_R)\,(h\,s-1)\,e-h\,s)\,\lambda\,(q\,\rho+\alpha)}{\lambda\,q+\alpha} \\ -\frac{\lambda\,(q\,\rho+\alpha)}{\lambda\,q+\alpha} \\ -\frac{\lambda\,(q\,\rho+\alpha)}{\lambda\,q+\alpha} \\ -\frac{\lambda\,(q\,\rho+\alpha)}{\lambda\,q+\alpha} \end{array}\right] \quad \textbf{(6)}$$

(7)

> *# We have a zero eigenvalue because of the line of Wildtype:Resistant equilibria. Four of the remaining five eigenvalues are negative by inspection because of demographic parameters. Final eigenvalue can have positive or negative sign, corresponding to invasion or loss of drive at this equilibrium:*

> $solve\left(\frac{((-1+q\_R)\,(h\,s-1)\,e-h\,s)\,\lambda\,(q\,\rho+\alpha)}{\lambda\,q+\alpha}=0, q\_R\right);$

$$\frac{e\,h\,s+h\,s-e}{(h\,s-1)\,e} \quad \textbf{(8)}$$

> *# This is the invasion threshold criterion*

>
